# Task-Dependent Functional and Effective Connectivity during Conceptual Processing

**DOI:** 10.1093/cercor/bhab026

**Published:** 2021-03-03

**Authors:** Philipp Kuhnke, Markus Kiefer, Gesa Hartwigsen

**Affiliations:** Lise Meitner Research Group Cognition and Plasticity, Max Planck Institute for Human Cognitive and Brain Sciences, Leipzig 04103, Germany; Department of Psychiatry, Ulm University, Ulm 89081, Germany; Lise Meitner Research Group Cognition and Plasticity, Max Planck Institute for Human Cognitive and Brain Sciences, Leipzig 04103, Germany

**Keywords:** DCM, grounded cognition, language, PPI, semantic memory

## Abstract

Conceptual knowledge is central to cognition. Previous neuroimaging research indicates that conceptual processing involves both modality-specific perceptual-motor areas and multimodal convergence zones. For example, our previous functional magnetic resonance imaging (fMRI) study revealed that both modality-specific and multimodal regions respond to sound and action features of concepts in a task-dependent fashion (Kuhnke P, Kiefer M, Hartwigsen G. 2020b. Task-dependent recruitment of modality-specific and multimodal regions during conceptual processing. Cereb Cortex. 30:3938–3959.). However, it remains unknown whether and how modality-specific and multimodal areas interact during conceptual tasks. Here, we asked 1) whether multimodal and modality-specific areas are functionally coupled during conceptual processing, 2) whether their coupling depends on the task, 3) whether information flows top-down, bottom-up or both, and 4) whether their coupling is behaviorally relevant. We combined psychophysiological interaction analyses with dynamic causal modeling on the fMRI data of our previous study. We found that functional coupling between multimodal and modality-specific areas strongly depended on the task, involved both top-down and bottom-up information flow, and predicted conceptually guided behavior. Notably, we also found coupling between different modality-specific areas and between different multimodal areas. These results suggest that functional coupling in the conceptual system is extensive, reciprocal, task-dependent, and behaviorally relevant. We propose a new model of the conceptual system that incorporates task-dependent functional interactions between modality-specific and multimodal areas.

## Introduction

Conceptual knowledge is crucial for many cognitive abilities, such as object recognition and use, as well as word comprehension (Lambon Ralph 2014; van Elk et al. 2014). Therefore, a central question in cognitive neuroscience has been how concepts are represented and processed in the human brain.

Research on the neural basis of conceptual processing has largely focused on functional segregation—identifying the different brain regions involved in conceptual processing and their functions. These studies have suggested that conceptual processing relies on both modality-specific perceptual-motor regions and cross-modal convergence zones (for reviews, see Kiefer and Pulvermüller 2012; Meteyard et al. 2012; Borghesani and Piazza 2017). Modality-specific regions represent perceptual-motor features of concepts. For example, action features are represented in motor and somatosensory regions (Hauk et al. 2004; Fernandino et al. 2016; Vukovic et al. 2017), whereas sound features are represented in auditory regions (Kiefer et al. 2008; Bonner and Grossman 2012). These findings support grounded cognition theories, which propose a functional-anatomical overlap between conceptual processing and real perceptual-motor experience (Barsalou 2008; Kiefer and Barsalou 2013). Cross-modal convergence zones, on the other hand, integrate modality-specific features into increasingly abstract representations (Damasio 1989; Simmons and Barsalou 2003; Binder 2016).

A common terminology is still lacking in the field and key terms (e.g., “modality”, “modality-specific”, and “cross-modal”) are widely used but rarely explicitly defined. However, these terms are useful to distinguish brain regions based on their representational abstraction from direct perceptual-motor experience (Binder 2016; Margulies et al. 2016). Therefore, we propose the following working definitions for this article: We refer to “perceptual-motor modalities” as the brain’s major input and output channels of perception and action (e.g., motor, somatosensory, auditory, visual, etc.). Note that these modalities do not simply correspond to the senses (hence the term “perceptual-motor” and not “sensory”) as they include channels of internal perception (e.g., emotion, proprioception) as well as motor action (Barsalou 2008; Kiefer and Barsalou 2013). Within the modalities, several dimensions can be further distinguished. For example, the visual modality includes the dimensions shape, color, motion, etc., which are processed by specialized neural circuits within the visual system (Van Essen and Maunsell 1983; Felleman and Van Essen 1991). We call brain regions “modality-specific” if they represent information related to a single perceptual-motor modality (Kiefer and Pulvermüller 2012). Regions are called “cross-modal” if they integrate information from at least two modalities into more abstract, cross-modal representations (Binder 2016).

We recently proposed a distinction among cross-modal convergence zones between “multimodal” areas that retain modality-specific information, and “amodal” areas that do not (Kuhnke et al. 2020b). That is, “amodal” regions contain the most abstract, modality-invariant conceptual representations, and are relevant for processing all types of conceptual information, regardless of perceptual-motor content (Jefferies 2013; Lambon Ralph et al. 2016). Previous evidence suggests that the left posterior parietal cortex (PPC) represents a “multimodal” convergence zone (Fernandino et al. 2016; Kuhnke et al. 2020b), whereas the anterior temporal lobe (ATL) acts as an “amodal” hub (Jefferies 2013; Lambon Ralph et al. 2016). For example, the left PPC responds to both sound and action features of concepts, whereas the ATL responds to general conceptual information (words > pseudowords) but not to modality-specific features (Kuhnke et al. 2020b; for similar results, see Fernandino et al. 2016). The amodal ATL appears to represent an abstract conceptual similarity structure that transcends individual modalities (Lambon Ralph et al. 2010; Patterson and Lambon Ralph 2016). Such an amodal conceptual representation seems necessary to explain the emergence of coherent conceptual categories (Lambon Ralph et al. 2010). In support of this view, evidence from semantic dementia (Patterson et al. 2007; Jefferies 2013), functional neuroimaging (Visser et al. 2010; Rice et al. 2015), transcranial magnetic stimulation (TMS; Pobric et al. 2010a, 2010b), and computational modeling (Rogers et al. 2004; Chen et al. 2017; Jackson et al. 2021) indicates a crucial role of the ATL in conceptual processing across virtually all types of concepts, regardless of their perceptual-motor content.

Overall, current evidence seems most consistent with “hybrid theories” that propose conceptual processing to rely on a representational hierarchy from modality-specific regions to multiple levels of cross-modal convergence zones (Binder and Desai 2011; Fernandino et al. 2016; Kiefer and Harpaintner 2020; Kuhnke et al. 2020b). Crucially, this hierarchical system is flexible, with different regions being recruited dynamically depending on the task (Hoenig et al. 2008; Kemmerer 2015; Popp et al. 2019b). For instance, both modality-specific and multimodal areas selectively respond to sound and action features when these are task-relevant (Kuhnke et al. 2020b).

However, little is known about functional integration within the conceptual system, that is, whether and how different regions interact during conceptual processing. Although some studies have investigated functional coupling between amodal ATL and modality-specific areas (Jackson et al. 2016; Chiou and Lambon Ralph 2019), it remains unknown whether and how multimodal areas (e.g., left PPC) interact with modality-specific regions. Here, we asked whether modality-specific and multimodal areas are coupled during conceptual processing, whether their coupling depends on the task, whether information flows bottom-up, top-down or bidirectionally, and whether their coupling is relevant for behavior.

We combined whole-brain, data-driven psychophysiological interaction (PPI) analyses with dynamic causal modeling (DCM) on the functional magnetic resonance imaging (fMRI) data of Kuhnke et al. (2020b). A total of 40 healthy participants performed three different tasks—lexical decision, sound judgment, and action judgment—on the same words with a high or low association to sounds and actions. PPI tested for task-dependent changes in functional coupling between modality-specific and multimodal seed regions with the rest of the brain (Friston et al. 1997; McLaren et al. 2012). As seed regions, we chose the somatomotor, auditory, and multimodal brain regions that exhibited the strongest functional activation for action knowledge retrieval, sound knowledge retrieval, or both, respectively (Kuhnke et al. 2020b). The results informed a DCM analysis that assessed the direction of information flow between multimodal and modality-specific areas (Kahan and Foltynie 2013; Zeidman et al. 2019a).

We hypothesized that modality-specific and multimodal areas interact in a task-dependent manner during conceptual processing. Multimodal regions should interact with somatomotor regions selectively during action feature retrieval and with auditory regions during sound feature retrieval. Based on previous work, we expected information to flow top-down (Damasio 1989; Fernandino et al. 2016) and bottom-up (Kiefer et al. 2011; Sim et al. 2015). Crucially, task-dependent functional coupling between modality-specific and multimodal areas should predict behavior in a modality-specific fashion: Interindividual differences in coupling between multimodal and somatomotor or auditory regions should correlate with personal action and sound associations, respectively.

## Materials and Methods

### Subjects

Data from 40 native German speakers [22 female; mean age: 26.6 years; standard deviation (SD): 4.1; range: 19–33] were analyzed. A total of 42 participants were initially recruited, but two were excluded due to strong head movement or aborting the experiment. All participants were right-handed (mean laterality quotient: 93.7; SD: 9.44; Oldfield 1971) and had no history of neurological disorders or head injury, or exhibited contraindications to fMRI. They were recruited via the subject database of the Max Planck Institute for Human Cognitive and Brain Sciences, Leipzig, Germany. Written informed consent was obtained from each subject prior to the experiment. The study was performed according to the guidelines of the Declaration of Helsinki and approved by the local ethics committee of the University of Leipzig.

### Experimental Procedures

**Figure 1:**
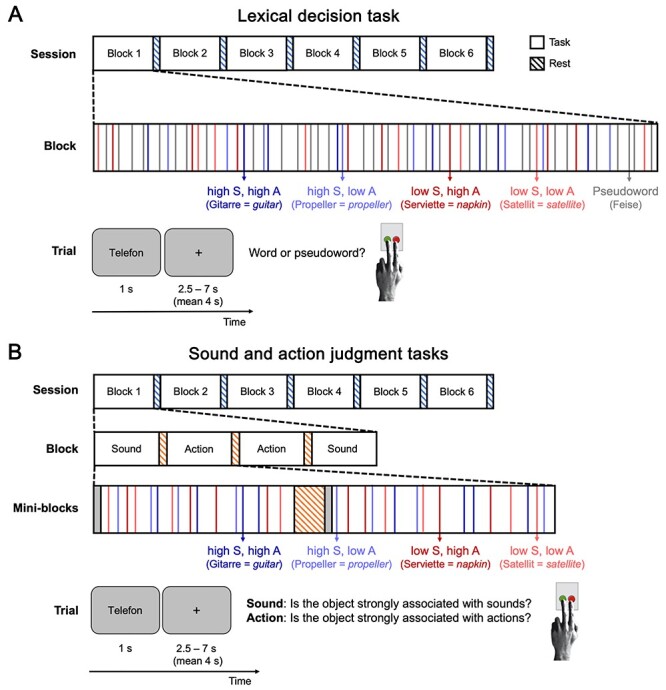
Experimental procedure. In two fMRI sessions, participants performed a lexical decision task (*A*), and sound and action judgment tasks (*B*). Trials for the four word types high sound–high action (dark blue), high sound–low action (light blue), low sound–high action (dark red), and low sound–low action (light red) were presented in random order within six blocks (64 trials each). Blocks were separated by 20-s rest periods (blue-striped bars). Sound and action judgment tasks were performed in mini-blocks of 16 trials, separated by 12-s rest periods (orange-striped bars). On each trial, a word was shown for 1 s, followed by an intertrial interval (fixation cross) of 2.5-7 s. Participants responded via button press.

The experimental procedure is reported in detail in Kuhnke et al. (2020b) and summarized here. The study employed a 3 × 2 × 2 within-subject design with the factors TASK (lexical decision, sound judgment, action judgment), SOUND (high, low association), and ACTION (high, low association). In two event-related fMRI sessions, participants performed three different tasks on 192 words with a high or low association to sounds and actions (Fig. 1). In the first session, participants performed a lexical decision task, in which they decided whether the presented stimulus was a real word or pseudoword. In the second session, participants performed sound and action judgment tasks. In the sound judgment task, participants judged whether the object denoted by the word was strongly associated with sounds. In the action judgment task, participants judged whether the object was strongly associated with actions. Whereas the lexical decision task acted as a control task that did not require sound or action knowledge, the sound and action judgment tasks explicitly required sound and action knowledge, respectively.

High and low sound words selectively differed in their association with sounds, whereas high and low action words differed only in their association with actions, as determined by the ratings of a different group of 163 volunteers (cf. Trumpp et al. 2014; Fernandino et al. 2016). Experimental conditions were matched on ratings of visual conceptual associations and familiarity, number of letters and syllables, word frequency, bi- and trigram frequencies, and number of orthographic neighbors (see the Supplementary Material of Kuhnke et al. 2020b). Stimuli for all conditions were selected from the same superordinate categories of animals, inanimate natural entities, and man-made objects (Goldberg 2006; Kiefer et al. 2008). For the lexical decision task, a pseudoword was generated for each word matched in length, syllable structure, and transition frequencies using the “Wuggy” software (Keuleers and Brysbaert 2010; http://crr.ugent.be/Wuggy).

At the end of the second session, we administered functional localizers for brain regions involved in auditory perception and somatomotor action. In the auditory localizer, participants attentively listened to 18-s blocks of real sounds, interspersed with 16-s silence blocks (cf. Kiefer et al. 2008; Hoenig et al. 2011). In the somatomotor localizer, participants performed different types of hand movements (finger tapping, fist making, pinching) in 18-s blocks, separated by 16-s rest blocks (cf. Bonner et al. 2013). Note that these localizers were designed to identify brain regions involved in real sound perception and somatomotor action, which may include areas beyond modality-specific circuits (see Discussion). Despite these limitations, the localizers allowed us to test the basic prediction of grounded cognition theories of a functional-anatomical overlap between conceptual processing and real perceptual-motor experience (cf. Kiefer et al. 2008; Hoenig et al. 2011; Hsu et al., 2011; Bonner et al. 2013).

### fMRI Data Acquisition and Preprocessing

fMRI data were collected on a 3 T Prisma scanner (Siemens, Erlangen, Germany) equipped with a 32-channel head coil. Functional, blood oxygenation level dependent (BOLD) images were acquired using a multiband dual gradient-echo echo-planar imaging (EPI) sequence [repetition time (TR): 2 s; echo times (TE): 12 and 33 ms; flip angle: 90°; field of view (FoV): 204 mm; voxel size: 2.5 × 2.5 × 2.5 mm; slice gap: 0.25 mm; bandwidth: 1966 Hz/Px; phase encoding direction: A/P]. A total of 60 slices covering the whole brain were recorded in interleaved order and axial orientation. We combined a multiband factor of 2 with in-plane GRAPPA acceleration of 2x (Feinberg et al. 2010), which exhibits a very low probability for false-positive activation due to slice leakage (Todd et al. 2016). We used a dual-echo sequence (Poser et al. 2006; Halai et al. 2014) and tilted slices 10° up (at the anterior edge) from the anterior commissure-posterior commissure line (Weiskopf et al. 2006) to minimize susceptibility artifacts and maximize BOLD sensitivity throughout the entire brain, including in regions suffering from signal loss in single-echo EPI such as the ATL (Devlin et al. 2000). B0 field maps were acquired for susceptibility distortion correction using a gradient-echo sequence (TR: 0.62 s; TE: 4 and 6.46 ms; flip angle: 60°; bandwidth: 412 Hz/Px; other parameters identical to functional sequence). Structural T1-weighted images were acquired for normalization using an MPRAGE sequence (176 slices in sagittal orientation; TR: 2.3 s; TE: 2.98 ms; FoV: 256 mm; voxel size: 1 × 1 × 1 mm; no slice gap; flip angle: 9°; phase encoding direction: A/P).

fMRI analysis was performed using Statistical Parametric Mapping (SPM12; Wellcome Trust Centre for Neuroimaging; http://www.fil.ion.ucl.ac.uk/spm/) implemented in Matlab (version 9.3). The two images with a short and long TE were combined using an average weighted by the temporal signal-to-noise ratio (tSNR) at each voxel, which yields optimal BOLD sensitivity (Poser et al. 2006). tSNR was calculated based on 30 volumes collected at the beginning of each scanning run, which were excluded from further analyses. Functional images were realigned, distortion corrected, slice-timing corrected, normalized to Montreal Neurological Institute (MNI) space, and smoothed with a 5 mm^3^ FWHM Gaussian kernel. An analysis of mean tSNR in anatomical regions-of-interest indicated satisfactory signal quality across the brain, including in the ATL (Supplementary Table 1).

### Psychophysiological Interactions

We leveraged the PPI approach to identify brain regions that show task-dependent functional coupling with auditory, somatomotor, and multimodal regions during conceptual processing. PPI reveals regions that exhibit task-dependent functional connectivity with a seed region-of-interest (ROI), above and beyond their task-independent connectivity (correlation), and task-related activation (O’Reilly et al. 2012). We employed generalized PPI (gPPI) that extends the PPI approach to experimental designs with more than two conditions (like the present one) for which standard SPM PPI is invalid (McLaren et al. 2012).

We used the group-constrained subject-specific approach (Julian et al. 2012) to define seed ROIs based on subject-specific functional activation (Fedorenko et al. 2010; Nieto-Castañón and Fedorenko 2012). This approach yields higher sensitivity and functional resolution (i.e., the ability to separate adjacent but functionally distinct regions) than the classical approach of defining ROIs based on the same location in standard space (Fedorenko and Kanwisher 2009, 2011; Nieto-Castañón and Fedorenko 2012). We defined three seed ROIs: 1) a “somatomotor seed”—the somatomotor region most strongly engaged in action feature retrieval—using the conjunction [Action judgment: high > low action words] ∩ [Somatomotor localizer: hand movements > rest]; 2) an “auditory seed”—the auditory region most strongly engaged in sound feature retrieval—using the conjunction [Sound judgment: high > low sound words] ∩ [Auditory localizer: real sounds > silence]; and 3) a “multimodal seed”—the brain region most strongly engaged in both action and sound feature retrieval—using the conjunction [Action judgment: high > low action words] ∩ [Sound judgment: high > low sound words] (for details on the activation analyses, see Kuhnke et al. 2020b). For each seed type, subject-specific activation maps were thresholded at *P* < 0.05 and overlaid on top of each other. The resulting overlap map was smoothed (5 mm), thresholded at two subjects, and parcellated using a watershed algorithm (Meyer 1991) implemented in the spm_ss toolbox (Nieto-Castañón and Fedorenko 2012). We retained the parcel with the strongest activation at the group level. Seed ROIs were then defined in each individual subject as the 10% most active voxels for the conceptual contrast within the parcel (Fedorenko et al. 2012; Basilakos et al. 2018).

We performed a whole-brain random-effects group analysis based on the general linear model (GLM), using the standard two-level approach. At the first level, individual participant data were modeled separately using the gPPI toolbox (version 13.1; https://www.nitrc.org/projects/gppi). The participant-level GLM included: 1) “Psychological” regressors for all experimental conditions, that is, stick functions at trial onsets convolved with the canonical hemodynamic response function (HRF). Only correct trials were included; error trials were modeled in a separate regressor of no interest. 2) A “physiological” regressor formed by the first eigenvariate of the seed ROI time series (i.e., the first principle component of the multivariate time series across all voxels in the ROI). 3) PPI regressors for each experimental condition created by multiplying the deconvolved BOLD signal of the seed ROI with the condition onsets and convolving with the canonical HRF (Gitelman et al. 2003; McLaren et al. 2012). 4) Nuisance regressors, including the six head motion parameters, individual regressors for time points with strong volume-to-volume movement (framewise displacement > 0.9; Siegel et al. 2014), and a duration-modulated parametric regressor accounting for response time differences between trials and conditions (Grinband et al. 2008).

Contrast images were computed for each participant and submitted to *t*-tests at the group level. To test for functional coupling during sound and action feature retrieval, we compared the connectivity for high > low sound words, and high > low action words within each task (lexical decision, sound judgment, action judgment). Conjunction analyses based on the minimum statistic (testing the conjunction null hypothesis; Nichols et al. 2005) tested for overlap between functional coupling for action or sound feature retrieval and activation in the somatomotor localizer (hand movements > rest) or auditory localizer (real sounds > silence), respectively. Finally, interaction analyses tested for task dependency in functional coupling by directly comparing the coupling increase for action features (high vs. low action words) or sound features (high vs. low sound words) between tasks (using paired *t*-tests). Interactions were inclusively masked by the minuend (within-task) contrast (Noppeney et al. 2006; Hardwick et al. 2018; Kuhnke et al. 2020b). For all group-level analyses, a gray matter mask was applied, restricting statistical tests to voxels with a gray matter probability > 0.3 (SPM12 tissue probability map). All activation maps were thresholded at a voxel-wise *P* < 0.001 and a cluster-wise *P* < 0.05 family-wise error (FWE) corrected for multiple comparisons.

To investigate whether task-dependent functional coupling between modality-specific and multimodal regions is relevant for behavior, we performed several PPI–behavior correlation analyses. To this end, we extracted the mean connectivity *t*-value of each participant from group-level PPI clusters (see Results section). We then performed Bayesian linear correlations between participants’ connectivity values and their personal action or sound ratings for the respective words. Ratings were collected outside the scanner after the fMRI measurements and reflected how strongly a participant personally associated each word with actions or sounds (on a 1-to-6 scale). A control analysis tested whether interindividual differences in sound or action ratings also correlated with response times in the sound and action judgment tasks for the same words. Bayesian correlation analyses were performed using the “JASP” program (https://jasp-stats.org/;Wagenmakers et al. 2018), and tested whether the data were better predicted by the null hypothesis (i.e., no correlation) or alternative hypothesis (i.e., positive correlation between functional coupling strength and individual ratings). BF_10_ denotes the Bayes Factor in favor of the alternative hypothesis, whereas BF_01_ refers to the Bayes Factor in favor of the null hypothesis (where BF_01_ = 1/BF_10_). For example, BF_10_ = 3 means that the data were three times more likely under the alternative hypothesis than under the null hypothesis (Lakens et al. 2020).

### Dynamic Causal Modeling

Although PPI can reveal task-dependent changes in functional coupling between a seed region and the rest of the brain, it cannot assess the direction of information flow between brain regions. Consequently, we additionally performed DCM (Friston et al. 2003) to assess directed causal influences between the network nodes identified in our PPI analyses. DCM estimates a model of effective connectivity between brain regions to predict a neuroimaging time series. A DCM consists of three types of parameters: 1) “intrinsic” (i.e., task-independent) directed connections between brain regions, 2) “modulatory inputs” that change connection strengths during a certain experimental manipulation, and 3) “driving inputs” that drive activity in the network. The goal of DCM is to optimize a tradeoff between model fit (of the predicted to observed time series) and complexity (i.e., deviation of model parameters from their prior expectations), measured by the model evidence (Kahan and Foltynie 2013; Zeidman et al. 2019a).

We performed a two-level analysis using parametric empirical bayes (PEB) and Bayesian model reduction (BMR)—the current “standard practice for group DCM studies” (Friston et al. 2016). At the first level, a “full model” was specified and estimated for each participant (see Results section). Regions included in the model were the left PPC (corresponding to the multimodal PPI seed), left auditory association cortex (AAC; group cluster from PPI analysis for sound feature retrieval), and left motor/somatosensory cortex (M1/S1; group cluster from PPI analysis for action feature retrieval). The first eigenvariate of the BOLD time series of each region was extracted and adjusted for effects-of-interest (all experimental conditions) using a GLM that modeled all trials as stick functions convolved with the canonical HRF, and regressed out the six motion parameters, high-movement time points (framewise displacement > 0.9; Siegel et al. 2014), and response time differences (Grinband et al. 2008). DCM inputs were mean-centered, so that the intrinsic connections reflected the mean connectivity across experimental conditions (Zeidman et al. 2019a).

At the second level, DCM parameters of individual participants were entered into a GLM—the PEB model—that decomposed interindividual variability in connection strengths into group effects and random effects (Zeidman et al. 2019b). BMR then compared the full model against numerous reduced models that had certain parameters “switched off” (i.e., prior mean and variance set to 0) (Friston et al. 2016). Finally, we computed the Bayesian model average (BMA), the average of parameter values across models weighted by each model’s posterior probability (*Pp*) (Penny et al. 2007). This approach is preferred over exclusively assessing the parameters of the “best” model as it accommodates uncertainty about the true underlying model (Friston et al. 2016; Dijkstra et al. 2017). The BMA was thresholded to only retain parameters with a *Pp* > 95% (Zeidman et al. 2019b). For each modulatory input, we calculated the resulting connectivity value (in Hz) using formula 3 in Zeidman et al. (2019a). Finally, to determine whether one experimental condition modulated a certain connection more strongly than another, we directly compared different modulatory inputs on the same connection using Bayesian contrasts (Dijkstra et al. 2017).

## Results

### Psychophysiological Interactions

We performed a PPI analysis to investigate task-dependent changes in functional coupling between modality-specific and multimodal “seed” regions with the rest of the brain during conceptual processing. We defined three seed regions: 1) a “somatomotor seed”—the motor region most strongly engaged in action feature retrieval, 2) an “auditory seed”—the auditory region most strongly engaged in sound feature retrieval, and 3) a “multimodal seed”—the brain region most strongly engaged in both action and sound feature retrieval. We identified the “somatomotor seed” in the left anterior inferior parietal lobe (aIPL)/primary somatosensory cortex (S1), the “auditory seed” in the left middle frontal gyrus (MFG)/precentral sulcus (PreCS), and the “multimodal seed” in the left PPC.

#### Somatomotor Seed (Left aIPL/S1)

**Figure 2:**
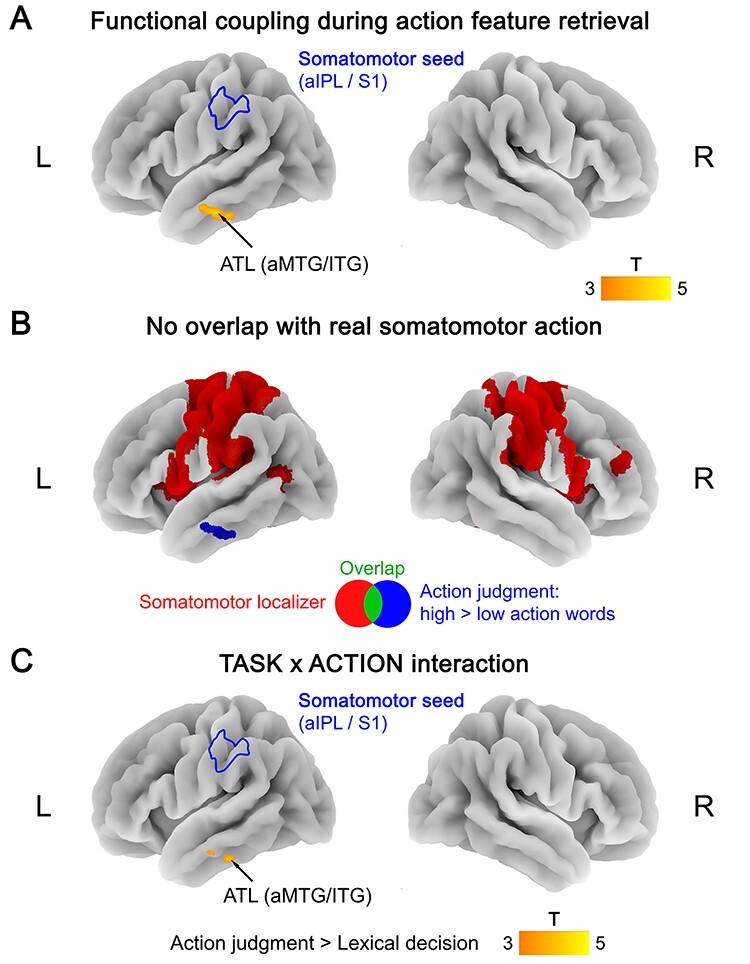
(*A*) Functional coupling with the somatomotor seed (left aIPL/S1) during action feature retrieval (action judgments: high > low action words). (*B*) No overlap between functional coupling with the somatomotor seed during action feature retrieval (blue) and activation for the somatomotor localizer (red; hand movements > rest). (*C*) TASK × ACTION interaction in functional coupling with the somatomotor seed, reflecting a stronger coupling increase for action features (high vs. low action words) during action judgments than lexical decisions. All statistical maps were thresholded at a voxel-wise *P* < 0.001 and a cluster-wise *P* < 0.05 FWE-corrected.

During action judgments, retrieval of action features (high > low action words) increased functional coupling between the somatomotor seed (left aIPL/S1) and the left ATL (including anterior middle and inferior temporal gyri) (Fig. 2*A*; Supplementary Table 2). The ATL region did not overlap with the somatomotor localizer (Fig. 2*B*), suggesting that it represents a higher-level, cross-modal area.

Functional coupling with the somatomotor seed was task-specific for action judgments. During sound judgments or lexical decisions, we found no increased coupling for action features (high > low action words) between the somatomotor seed and any other brain region. In addition, interaction analyses revealed a TASK × ACTION interaction in functional coupling with the somatomotor seed: Left ATL showed a stronger coupling increase for action features (high vs. low action words) during action judgments than during lexical decisions (Fig. 2*C*; Supplementary Table 3).

Moreover, the functional connectivity change was specific to action features: No region showed significant functional coupling with the somatomotor seed for sound features (high > low sound words) in any task.

#### Auditory Seed (Left MFG/PreCS)

**Figure 3:**
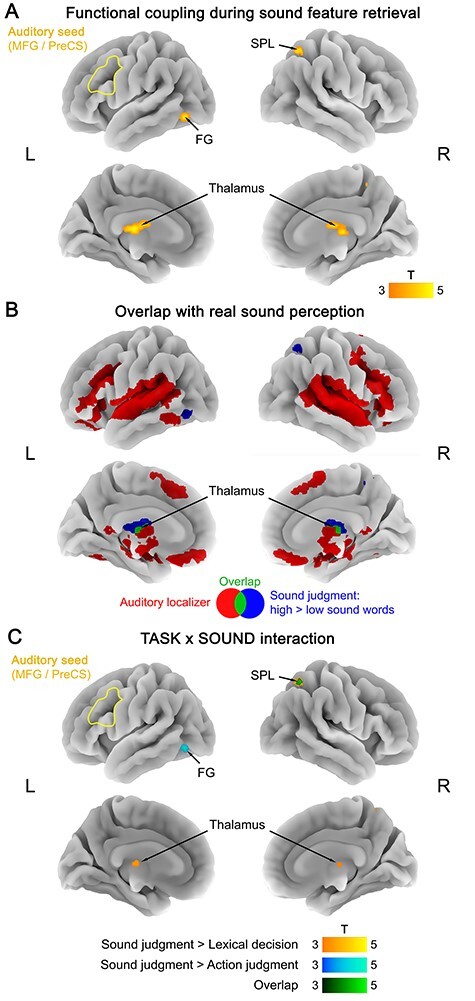
(*A*) Functional coupling with the auditory seed (left MFG/PreCS) during sound feature retrieval (sound judgments: high > low sound words). (*B*) Overlap (green) between functional coupling with the auditory seed during sound feature retrieval (blue) and activation for the auditory localizer (red; real sounds > silence). (*C*) TASK × SOUND interaction in functional coupling with the auditory seed, reflecting a stronger coupling increase for sound features (high vs. low sound words) during sound judgments than during lexical decisions (yellow), action judgments (blue), or both (green). All statistical maps were thresholded at a voxel-wise *P* < 0.001 and a cluster-wise *P* < 0.05 FWE-corrected.

During sound judgments, retrieval of sound features (high > low sound words) increased functional connectivity between the auditory seed (left MFG/PreCS) and the thalamus, left fusiform gyrus (FG), and right superior parietal lobe (SPL) (Fig. 3*A*; Supplementary Table 4*A*). The thalamus cluster partially overlapped with the auditory localizer (Fig. 3*B*; Supplementary Table 4*B*), indicating that it is involved in real sound perception. FG and SPL did not overlap with the auditory localizer.

Functional coupling with the auditory seed was task-specific for sound judgments. During action judgments or lexical decisions, we found no significant coupling changes between the auditory seed and any other brain region. In addition, interaction analyses revealed a TASK × SOUND interaction in functional coupling with the auditory seed: All three regions (thalamus, FG, SPL) showed a stronger coupling increase for sound features (high vs. low sound words) during sound judgments than during lexical decisions and/or action judgments (Fig. 3*C*; Supplementary Table 5).

Moreover, functional coupling with the auditory seed was specific to sound features. The auditory seed did not show increased coupling for action features (high > low action words) with any other brain area in any task.

#### Multimodal Seed (Left PPC)

**Figure 4:**
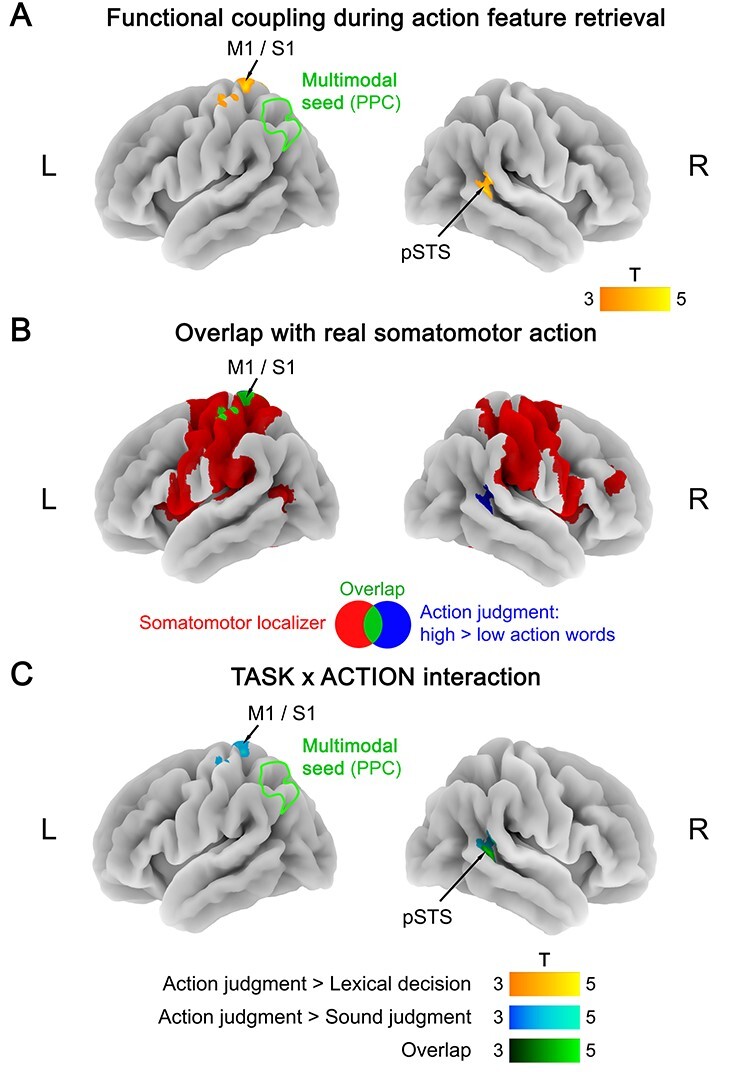
(*A*) Functional coupling with the multimodal seed (left PPC) during action feature retrieval (action judgments: high > low action words). (*B*) Overlap (green) between functional coupling with the multimodal seed during action feature retrieval (blue) and activation for the somatomotor localizer (red; hand movements > rest). (*C*) TASK × ACTION interaction in functional coupling with the multimodal seed, reflecting a stronger coupling increase for action features (high vs. low action words) during action judgments than during lexical decisions (yellow), sound judgments (blue), or both (green). All statistical maps were thresholded at a voxel-wise *P* < 0.001 and a cluster-wise *P* < 0.05 FWE-corrected.

The multimodal seed (left PPC) showed a double dissociation in its functional connectivity profile. During action judgments, retrieval of action features (high > low action words) selectively increased functional connectivity between the multimodal seed and left primary motor/somatosensory cortex (M1/S1; extending into SPL), as well as the right posterior superior temporal sulcus (pSTS) (Fig. 4*A*; Supplementary Table 6*A*). Left M1/S1 overlapped with the somatomotor localizer, whereas right pSTS did not (Fig. 4*B*; Supplementary Table 6*B*). Interaction analyses showed that both areas exhibited a TASK × ACTION interaction, driven by a larger coupling increase for action features (high vs. low action words) during action judgments than during lexical decisions and/or sound judgments (Fig. 4*C*; Supplementary Table 7). Sound features (high > low sound words) did not induce significant functional connectivity changes during action judgments.

**Figure 5:**
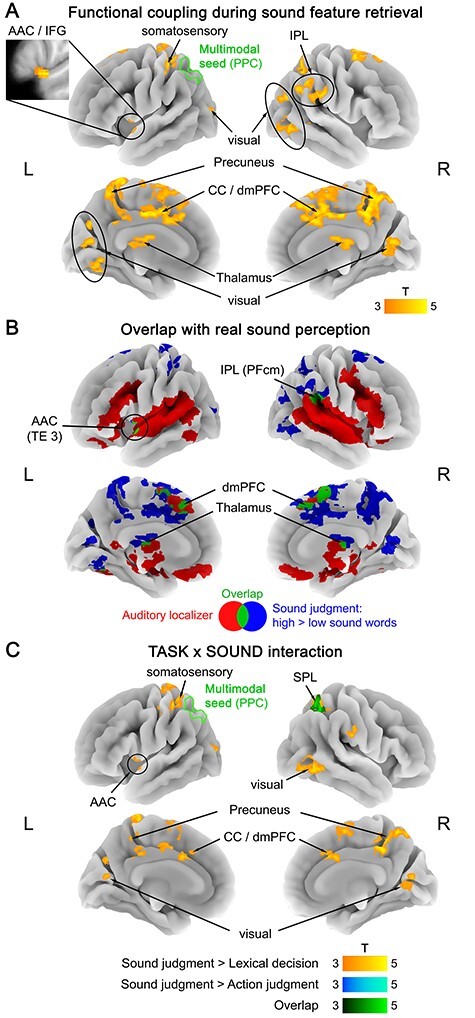
(*A*) Functional coupling with the multimodal seed (left PPC) during sound feature retrieval (sound judgments: high > low sound words). (*B*) Overlap (green) between functional coupling with the multimodal seed during sound feature retrieval (blue) and activation for the auditory localizer (red; real sounds > silence). (*C*) TASK × SOUND interaction in functional coupling with the multimodal seed, reflecting a stronger coupling increase for sound features (high vs. low sound words) during sound judgments than during lexical decisions (yellow), action judgments (blue), or both (green). All statistical maps were thresholded at a voxel-wise *P* < 0.001 and a cluster-wise *P* < 0.05 FWE-corrected.

Conversely, during sound judgments, sound feature retrieval (high > low sound words) increased functional connectivity between the multimodal seed and an extensive network of other brain regions (Fig. 5*A*; Supplementary Table 8*A*). Several of these regions overlapped with the auditory localizer (Fig. 5*B*; Supplementary Table 8*B*), including left AAC (extending into inferior frontal gyrus), right IPL, as well as bilateral dorsomedial prefrontal cortex (dmPFC) and thalamus. However, we also found increased functional coupling between the multimodal seed and several regions outside the auditory system, including bilateral precuneus, middle cingulate cortex, early visual cortex, and left somatosensory cortex. Most of these areas exhibited a TASK × SOUND interaction, driven by a stronger coupling increase for sound features (high vs. low sound words) during sound judgments than during lexical decisions and/or action judgments (Fig. 5*C*; Supplementary Table 9). No coupling changes were found for action features (high > low action words) during sound judgments.

During lexical decisions, we did not identify significant coupling changes with the multimodal seed, neither for action features (high > low action words) nor for sound features (high > low sound words).

#### Amodal seed (Left ATL)

As the ATL is widely considered a central, amodal hub of the conceptual system, we performed a supplementary PPI analysis seeding in the ATL (see Supplementary Material). This “amodal seed” (left ATL) showed a similar task-dependent double dissociation in functional coupling as the multimodal PPC. During sound judgments, left ATL showed increased coupling for sound features with the bilateral precuneus/posterior cingulate cortex (PC/PCC) (Supplementary Fig. 1; Supplementary Tables 10 and 11). During action judgments, left ATL exhibited increased coupling for action features with the left dmPFC (Supplementary Fig. 2; Supplementary Tables 12 and 13). Neither of these regions overlapped with the relevant perceptual-motor localizers. Therefore, whereas multimodal PPC interacted with modality-specific perceptual-motor regions, amodal ATL was functionally coupled with other high-level cross-modal convergence zones in a task-dependent fashion.

#### Functional Coupling between Multimodal and Modality-Specific Areas Is Relevant for Behavior

The PPI analyses identified networks of brain regions that interact with each other in a task-dependent manner during conceptual processing. Most strikingly, the multimodal region in left PPC functionally coupled with left AAC selectively during sound feature retrieval, and with left M1/S1 selectively during action feature retrieval. It remains unclear, however, whether these functional interactions are relevant for behavior. We reasoned that if the task-dependent functional coupling between multimodal left PPC and somatomotor or auditory cortex is behaviorally relevant, a participant’s individual coupling strength should be related to their personal action and sound associations with concepts. Crucially, this relationship should be modality-specific: Coupling between left PPC and M1/S1 (during action feature retrieval) should correlate with action, but not sound associations, whereas coupling between left PPC and AAC (during sound feature retrieval) should correlate with sound, but not action associations.

**Figure 6:**
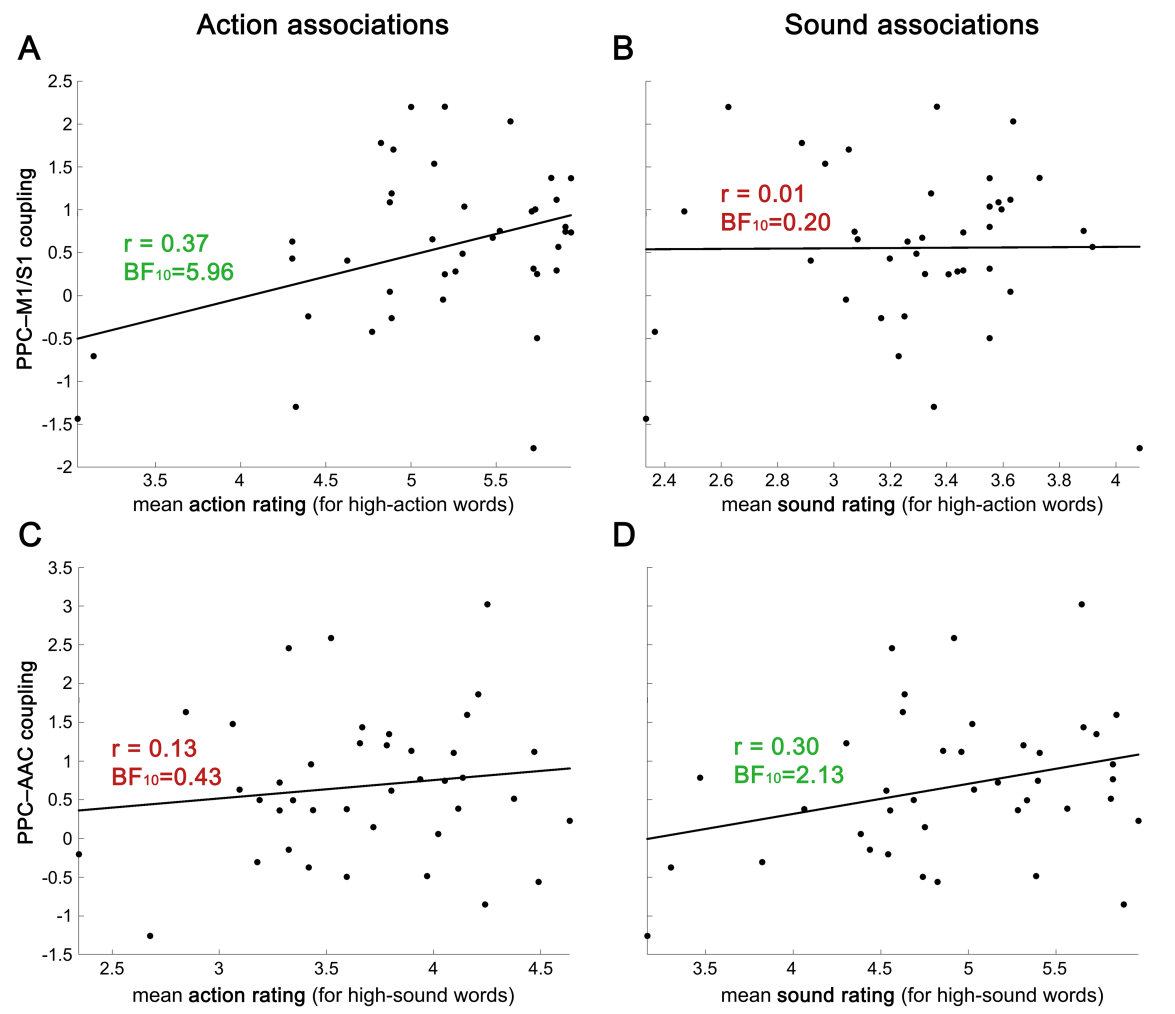
Individual functional coupling between multimodal left PPC and M1/S1 during action knowledge retrieval (PPI *t*-value for high > low action words during action judgments) predicted participants’ personal action associations (*A*) but not sound associations (*B*) for action-related words. Conversely, functional coupling between PPC and AAC during sound feature retrieval (PPI *t*-value for high > low sound words during sound judgments) correlated with participants’ individual sound associations (*D*) but not action associations (*C*) for sound-related words.

Indeed, we found that participants’ functional coupling strength between left PPC and M1/S1 for action-related (vs. unrelated) words during action judgments positively correlated with their personal action ratings for these words (Fig. 6*A*), but not with their sound ratings (Fig. 6*B*). For action ratings, the data were ~6 times more likely under the hypothesis that participants with stronger functional coupling between left PPC and M1/S1 during action feature retrieval had stronger action associations than under the null hypothesis of no correlation (BF_10_ = 5.96). For sound ratings, the data were ~5 times more likely under the null hypothesis (BF_10_ = 0.20 or equivalently BF_01_ = 4.91).

Conversely, the individual functional connectivity between left PPC and AAC for sound-related (vs. unrelated) words during sound judgments was associated with participants’ sound ratings (Fig. 6*D*; BF_10_ = 2.13), but not with their action ratings (Fig. 6*C*; BF_10_ = 0.43 or BF_01_ = 2.34). Thus, participants with stronger functional connectivity between left PPC and AAC had stronger sound associations for sound-related concepts. These results support the hypothesized modality-specific association between task-dependent functional coupling of multimodal with perceptual-motor brain areas and conceptual associations on the behavioral level.

A control analysis showed that action and sound ratings did not correlate with response times for action or sound judgments on the same words (Supplementary Fig. 3). Moreover, our PPI analyses included participant-specific response time regressors. This indicates that interindividual differences in action and sound conceptual associations, and their association with functional coupling between multimodal and modality-specific areas, cannot be explained by differences in action and sound judgment performance. Stronger functional coupling between multimodal PPC and somatomotor or auditory cortices predicts stronger action and sound conceptual associations, above and beyond task performance differences.

### Dynamic Causal Modeling

Although PPI can reveal task-dependent changes in functional coupling between a seed region and the rest of the brain, it cannot assess the direction of information flow between brain regions. To provide insight into the information flow between multimodal PPC and modality-specific areas, we leveraged the PPI results to inform a complementary DCM analysis (Friston et al. 2003). The DCM model included left PPC (the multimodal PPI seed), auditory cortex (AAC; PPI cluster for sound feature retrieval), and somatomotor cortex (M1/S1; PPI cluster for action feature retrieval). This analysis allowed us to determine whether information flow between multimodal and modality-specific areas is top-down, bottom-up, or bidirectional; and how it is modulated during sound and action knowledge retrieval.

**Figure 7:**
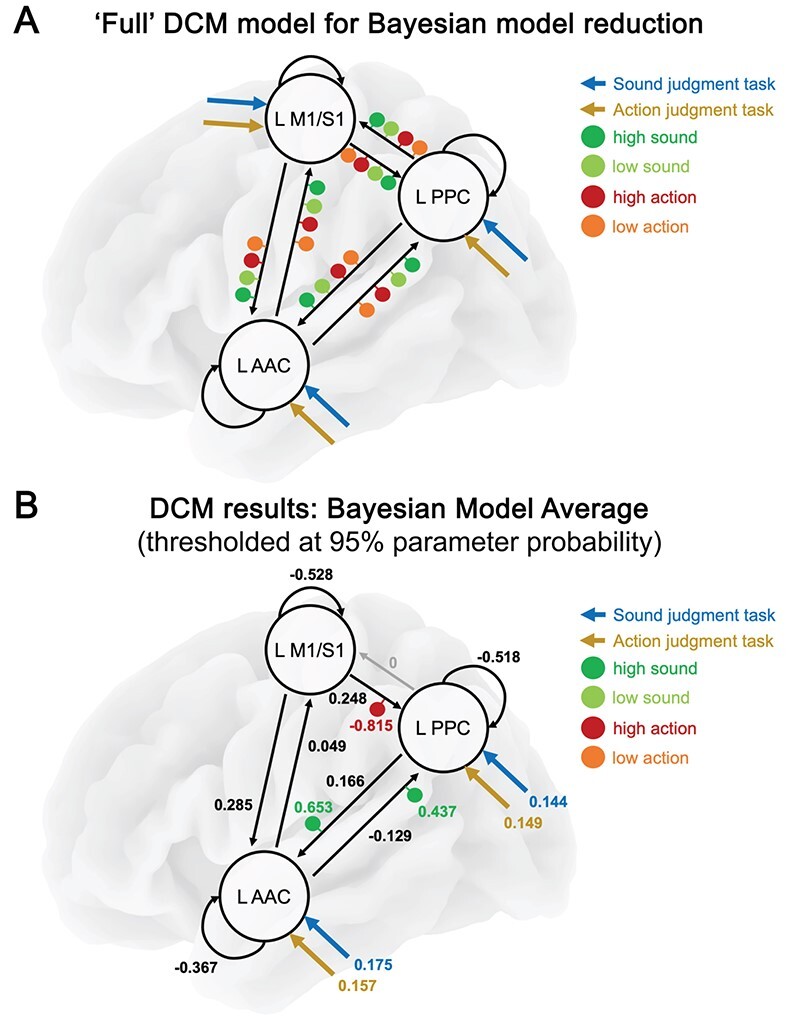
(*A*) The “full” DCM model that served as starting point for Bayesian model reduction. Black arrows represent intrinsic (i.e., task-independent) connections, colored arrows denote driving inputs (tasks), and colored dots represent modulatory inputs (word types). (*B*) The resulting BMA thresholded at 95% parameter probability. Driving and between-region parameters are in units of Hz. Modulatory parameters in- or decrease between-region parameters in an additive manner.

We performed a DCM group analysis using BMR (Friston et al. 2016; Zeidman et al. 2019b). To this end, a “full” DCM model was defined for each participant (Fig. 7*A*): In this model, left PPC, AAC, and M1/S1 were bidirectionally connected with each other. Sound and action judgment tasks could serve as driving inputs to every region. Each between-region connection could receive modulatory input from high- and low-sound words, as well as high- and low-action words.

BMR then compared this model with numerous reduced models that had certain parameters (e.g., connections, modulatory inputs) removed. Finally, we computed the Bayesian model average (BMA), the average of parameter values across models weighted by each model’s probability, and thresholded the BMA at 95% parameter probability. The results are shown in Fig. 7*B* and Table 1.

**Table 1:** Parameter estimates of the BMA

Connection	Intrinsic connectivity	*Pp*	high sound	*Pp*	low sound	*Pp*	high action	*Pp*	low action	*Pp*
PPC ➔ M1/S1	0.0 (0)	0.0	0.0 (0)	0.0	0.0 (0)	0.0	0.0 (0)	0.0	0.0 (0)	0.0
PPC ➔ AAC	**0.166 (0.001)**	**1.0**	**0.653 (0.020)**	**1.0**	0.0 (0)	0.0	0.0 (0)	0.0	0.0 (0)	0.0
M1/S1 ➔ PPC	**0.248 (0.001)**	**1.0**	0.0 (0)	0.0	0.470 (0.053)	0.92	**−0.815 (0.024)**	**1.0**	0.0 (0)	0.0
M1/S1 ➔ AAC	**0.258 (0.001)**	**1.0**	0.0 (0)	0.0	0.0 (0)	0.0	−0.200 (0.030)	0.67	0.0 (0)	0.0
AAC ➔ PPC	**−0.129 (0)**	**1.0**	**0.437 (0.014)**	**0.997**	0.0 (0)	0.0	0.0 (0)	0.0	0.0 (0)	0.0
AAC ➔ M1/S1	**0.049 (0)**	**0.999**	0.0 (0)	0.0	0.0 (0)	0.0	0.0 (0)	0.0	0.0 (0)	0.0

Note: Parameter covariance is given in parentheses. Bold font highlights parameters with a *Pp* > 95%.

#### Intrinsic Connectivity

We found strong evidence for all possible intrinsic (i.e., task-independent) connections between the three regions (*Pp* > 0.999), except for the connection from PPC to M1/S1 (*Pp* < 0.001). PPC had an excitatory connection to AAC; AAC weakly excited M1/S1 and inhibited PPC; and M1/S1 positively drove both PPC and AAC (Table 1).

#### Driving Inputs

Sound and action judgment tasks drove activity in PPC (sound: 0.144 Hz; action: 0.149 Hz) and AAC (sound: 0.175 Hz; action: 0.157 Hz), but not M1/S1. Importantly, Bayesian contrasts revealed that AAC was more strongly driven by sound than action judgments (*Pp* = 0.95), whereas left PPC was similarly driven by sound and action judgments (*Pp* = 0.79).

#### Modulatory Inputs

High-sound words selectively modulated reciprocal connectivity between PPC and AAC, further increasing the positive PPC-to-AAC connection (modulation: 0.653; result: 0.768 Hz), and even turning the negative AAC-to-PPC connection positive (modulation: 0.437; result: 0.275 Hz). Bayesian contrasts provided strong evidence that high-sound words modulated both connections more strongly than all other word types (vs. low-sound: *Pp* > 0.999; vs. high-action: *Pp* > 0.999; vs. low-action: *Pp* > 0.999), which showed a very low probability of modulating either connection (low-sound: *Pp* < 0.001; high-action: *Pp* < 0.001; high-sound: *Pp* < 0.001).

In contrast, high-action words selectively modulated the M1/S1-to-PPC connection, rendering the positive connection negative (modulation: −0.815; result: −0.539 Hz). High-action words modulated this connection more strongly than all other word types (vs. low-action: *Pp* > 0.999; vs. high-sound: *Pp* > 0.999; vs. low-sound: *Pp* > 0.999), which had a very low probability of modulation (low-action: *Pp* < 0.001; high-sound: *Pp* < 0.001; low-sound: *Pp* < 0.001). Low-action and low-sound words did not modulate any connection with a high probability.

## Discussion

This study investigated task-dependent functional and effective connectivity during conceptual processing. Specifically, we asked 1) whether modality-specific and multimodal areas interact during sound and action knowledge retrieval, 2) whether their coupling depends on the task, 3) whether information flows bottom-up, top-down, or bidirectionally, and 4) whether their coupling is relevant for behavior. Combining a whole-brain connectivity approach with directional effective connectivity analysis, we found that functional coupling between modality-specific and multimodal areas strongly depended on the task, involved both bottom-up and top-down information flow, and was behaviorally relevant: The multimodal region in the left posterior parietal cortex (PPC) showed increased coupling with left primary motor and somatosensory cortices (M1/S1) selectively when action knowledge was task-relevant. Conversely, multimodal PPC increased its functional interaction with left auditory association cortex (AAC) selectively when sound knowledge was task-relevant. DCM analyses further revealed that multimodal PPC was bidirectionally connected with AAC, and sound knowledge modulated both the top-down and bottom-up connections. In contrast, M1/S1 was unidirectionally connected to PPC, and action knowledge specifically modulated this bottom-up connection. Finally, coupling between multimodal PPC and somatomotor or auditory cortices predicted participants’ personal action and sound associations with concepts, respectively. This indicates that flexible connectivity between multimodal and modality-specific areas is crucial for conceptually guided behavior.

### Multimodal PPC vs. Amodal ATL

Our findings suggest that the multimodal region in left PPC acts as a functional coupling “switchboard” (cf. Wang et al. 2017; Chiou and Lambon Ralph 2019), flexibly adapting its connectivity to task-relevant modality-specific nodes. A similar function has recently been proposed for the ATL (Chiou and Lambon Ralph 2019). In that study, left ATL functionally coupled with motor regions during the implicit processing of action knowledge, and with place-related regions during the processing of place knowledge associated with object pictures. Consequently, these authors highlighted the importance of flexible coupling between the ATL and modality-specific regions during conceptual processing (see also Jackson et al. 2016; Lambon Ralph et al. 2016; Wang et al. 2017). However, based on our findings, we propose that the ATL is not unique in its role as a key node for flexible coupling with modality-specific regions, but left PPC plays a similar role in conceptual processing. This is in line with a graph-theoretic fMRI study that showed that left PPC and ATL exhibit particularly flexible functional connectivity during language processing, coactivating with different regions at different times (Chai et al. 2016).

Crucially, however, we propose a functional distinction between left PPC and ATL. In our previous fMRI study, left PPC was recruited for both sound and action features when they were task-relevant, responding to sound features during sound judgments and to action features during action judgments (Kuhnke et al. 2020b). In contrast, the ATL responded to general conceptual information (words > pseudowords; cf. Binder et al. 2009), but not to modality-specific features. These results suggest that left PPC is “multimodal” (i.e., sensitive to modality-specific information), whereas the ATL is “amodal” (i.e., insensitive to modality-specific information). This view is supported by another fMRI study that demonstrated that functional activation for word concreteness judgments correlated with the ratings for several perceptual-motor attributes in the PPC (and in mPFC and PC/PCC), but not in the ATL (Fernandino et al. 2016). Notably, we recently found that TMS over left PPC impairs behavioral performance for action, but not sound knowledge (Kuhnke et al. 2020a). Although these findings suggest that left PPC selectively supports action knowledge retrieval, they do not preclude an additional role of this area in sound knowledge retrieval. In particular, other sound-related regions may have compensated for the disruption of left PPC. Such compensatory mechanisms could be further investigated in future studies employing combined TMS-fMRI (Hartwigsen 2018).

Importantly, not only the regional response, but also the functional coupling profile seems to differ between multimodal PPC and amodal ATL. Indeed, a supplementary PPI analysis seeding in left ATL revealed task-dependent functional coupling with other high-level cross-modal regions, but not modality-specific cortices: Amodal ATL interacted with bilateral PC/PCC during sound feature retrieval (Supplementary Fig. 1), and with left dmPFC during action feature retrieval (Supplementary Fig. 2), neither of which overlapped with the relevant perceptual-motor localizers. Therefore, whereas multimodal PPC directly couples with modality-specific regions (e.g., left AAC and M1/S1), amodal ATL seems to mainly interact with other cross-modal convergence zones. Indeed, it might be exactly this difference in connectivity profiles that yields the difference in regional response profiles (cf. Lambon Ralph et al. 2016): Multimodal areas may be sensitive to modality-specific information by virtue of their direct interactions with modality-specific cortices. In contrast, amodal ATL might be insensitive to modality-specific features, as it exhibits little coupling with modality-specific areas.

Notably, we did observe coupling between the “somatomotor seed” in left aIPL/S1 and left lateral ATL (aMTG/ITG) during action feature retrieval (see Fig. 2). In contrast, our “amodal seed” in left ATL (defined using the contrast words > pseudowords) picked out functionally distinct voxels that selectively coupled with cross-modal, but not modality-specific nodes. This suggests that our “amodal seed” within left ATL was genuinely amodal, whereas the lateral ATL seemed to be biased toward action knowledge and connected with somatomotor areas. This dissociation is in line with the proposal of a “graded” modality-specificity within the ATL, which depends on the connectivity of different ATL subregions with modality-specific cortices (Pulvermüller et al. 2010; Lambon Ralph et al. 2016).

### Functional Coupling During Conceptual Processing is Extensive

In addition to task-dependent coupling between modality-specific and multimodal areas, PPI also revealed lateral connections between different modality-specific areas and between different multimodal areas. During sound knowledge retrieval, the auditory seed in left MFG/PreCS coupled with an auditory region in the thalamus, and multimodal PPC coupled with other multimodal areas in the PC/PCC and mPFC (Fernandino et al. 2016; Kuhnke et al. 2020b). Together with our DCM results, these findings indicate that functional coupling in the conceptual system is more extensive and reciprocal than previously thought. Specifically, our results conflict with the common view that concept retrieval relies mainly on top-down information flow from cross-modal to modality-specific areas (Damasio 1989; Meyer and Damasio 2009; Fernandino et al. 2016). Sound knowledge retrieval involved bidirectional coupling between multimodal PPC and AAC, and action knowledge retrieval even selectively relied on bottom-up input from primary motor/somatosensory cortex to multimodal PPC (cf. Kiefer et al. 2011; Sim et al. 2015).

Two additional findings are noteworthy. Firstly, during sound feature retrieval, we found evidence for coupling with nonauditory modality-specific regions. Auditory seed MFG/PreCS coupled with visual (FG) and somatomotor (SPL) areas, and multimodal PPC coupled with somatosensory and visual cortices (see Fig. 5). This “cross-modality coupling” might reflect that retrieval of sound features of an object (e.g., guitar) can coactivate its visual form, action and touch information, corroborating previous findings for functional activation (Reilly et al. 2016; Lemaitre et al. 2018; Popp et al. 2019b). Secondly, we found that functional coupling during conceptual knowledge retrieval involved low-level sensory-motor areas. Selectively during sound feature retrieval, a region of the thalamus activated in the auditory localizer showed increased coupling with both auditory MFG/PreCS and multimodal PPC. Although a precise anatomical localization is limited by our fMRI protocol, this thalamic area might reflect the medial geniculate nucleus, a low-level auditory region that even precedes primary auditory cortex in the auditory processing hierarchy (Henkel 2018). Moreover, during action feature retrieval, primary motor/somatosensory cortex interacted with multimodal PPC. Critically, low-level sensory-motor areas rarely show functional activation in conceptual tasks (Thompson-Schill 2003; Fernandino et al. 2016; but see Hauk et al. 2004; Harpaintner et al. 2020). Indeed, our activation analyses of the same data did not identify low-level sensory-motor activity (Kuhnke et al. 2020b). Such results led some authors to conclude that low-level sensory-motor areas are not involved in conceptual processing (Martin 2016). The present results question this view, suggesting that low-level areas can be involved, at least by influencing the activity of higher-level cortical areas. As a potential explanation for the discrepancy between functional activation and coupling, local activation is generally assumed to reflect intracortical synaptic processing of inputs, whereas connectivity changes reflect cortical outputs to functionally connected areas (Ward et al. 2010; Fiori et al. 2018).

### Involvement of Modality-Specific Perceptual-Motor Regions

To determine modality-specific perceptual-motor regions, we tested for overlap with activation during somatomotor and auditory localizers in the same participants. In the somatomotor localizer, participants performed different types of hand movements (finger tapping, pinching, fist making; cf. Bonner et al. 2013). Notably, the localizer itself was not modality-specific, involving both motor and somatosensory activity (due to somatosensory feedback during movement). However, it engaged modality-specific brain regions, such as primary motor cortex (M1) and primary somatosensory cortex (S1). Crucially, both motor and somatosensory areas are involved in object-directed actions (van Elk et al. 2014; Hardwick et al. 2018) as well as action-related conceptual processing (Desai et al. 2010; Fernandino et al. 2016; Kuhnke et al. 2020b). In our study, action feature retrieval involved coupling with both left M1 and S1. Importantly, left M1 and S1 were specifically involved in action, but not sound knowledge retrieval. Note that motor and somatosensory areas may play distinct roles within action knowledge processing, representing the movement versus touch-related components of object-directed actions, respectively. Future studies should aim to disentangle these motor and somatosensory components.

In the auditory localizer, participants listened to real object sounds. We presented meaningful object sounds, and not meaningless tones, as sound features of concepts should comprise high-level auditory information (e.g., barking; Bizley and Cohen 2013), rather than low-level acoustic information (e.g., loudness, pitch) (see also Kiefer et al. 2008; Hoenig et al. 2011). The use of real object sounds risks the concomitant engagement of (possibly amodal) conceptual representations (Simanova et al. 2014). Indeed, some regions engaged by the auditory localizer may be involved in abstract conceptual processing, rather than sound perception (e.g., bilateral dmPFC; Binder and Desai 2011; Binder and Fernandino 2015). However, our main conclusions regarding left AAC and thalamus are not compromised by this issue. Left AAC was determined cytoarchitectonically as region TE 3, which is part of high-level auditory cortex (Morosan et al. 2005; Bola et al. 2017). The thalamus is a low-level sensory region (Henkel 2018), unlikely to house amodal conceptual representations. Moreover, both regions were selectively involved in sound, but not action knowledge retrieval.

Overall, the localizers served to constrain our analyses and interpretations by identifying brain regions involved in somatomotor action and sound perception with a high sensitivity but low specificity. They were not designed to define modality-specific regions on their own. Rather, the combined evidence from connectivity profiles, perceptual-motor localizer overlap, and anatomical information suggests that action and sound feature retrieval involved functional coupling with modality-specific perceptual-motor regions.

In general, we observed a task-dependent dissociation between functional coupling during sound versus action knowledge retrieval. Sound features (high > low sound words) increased functional coupling selectively during sound judgments, whereas action features (high > low action words) increased coupling specifically during action judgments. These findings support the view that conceptual processing relies on a flexible, task-dependent architecture (Hoenig et al. 2008; Binder and Desai 2011; Kemmerer 2015; Popp et al. 2019a). Different features of a concept are selectively retrieved when they are task-relevant (Lebois et al. 2015; Yee and Thompson-Schill 2016). Note that differences between the lexical decision task and other tasks could be influenced by differences in session order or responses as lexical decisions were always performed first, and participants responded “yes” to all words. Importantly, however, the dissociation between sound and action judgments cannot be explained by order or response effects as these tasks were counterbalanced within and across participants, and the comparison of high versus low sound/action words corresponded to “yes” versus “no” responses in both cases.

**Figure 8:**
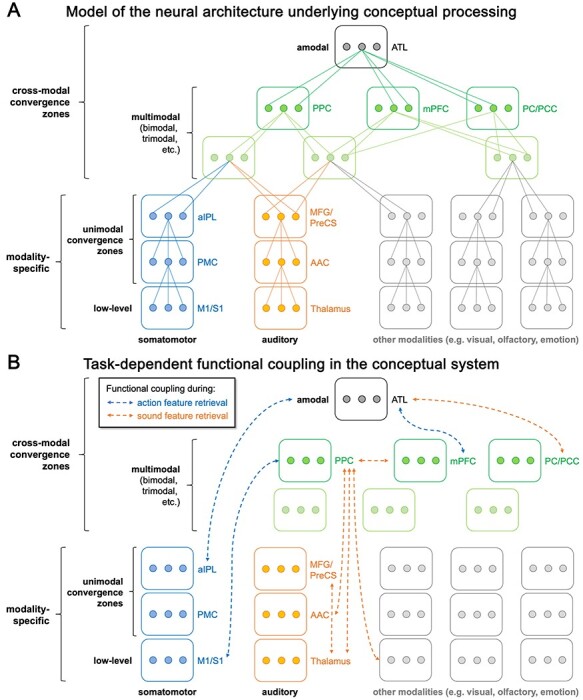
(*A*) A novel model of the neural architecture underlying conceptual processing. Low-level modality-specific representations converge onto more abstract modality-specific representations in unimodal convergence zones. Multimodal convergence zones integrate information across modalities, while retaining modality-specific information. Finally, amodal areas completely abstract away from modality-specific content. Boxes represent brain regions and connected dots represent individual representational units that converge onto a more abstract representation at a higher level. (*B*) Task-dependent functional coupling during action and sound feature retrieval. Functional coupling in the conceptual system is extensive and flexible. Modality-specific regions selectively come into play when the knowledge they represent is task-relevant. Multimodal PPC dynamically adapts its connectivity profile to task-relevant modality-specific nodes. Amodal ATL mainly interacts with other high-level cross-modal convergence zones in a task-dependent fashion.

### Future Directions to Study Functional and Effective Connectivity during Conceptual Processing

In our two-step analysis approach, we informed DCM with the results of a whole-brain PPI analysis on fMRI data. Crucially, DCM has been validated for face validity (i.e., confirming appropriate responses using simulated data; Friston et al. 2003; Stephan et al. 2009), construct validity (i.e., testing whether DCM is consistent with other approaches; Penny et al. 2004; Lee et al. 2006), predictive validity (i.e., testing whether DCM predicts a known or expected effect; David et al. 2008; Reyt et al. 2010), test–retest reliability (Schuyler et al. 2010) and reproducibility (Bernal-Casas et al. 2013). Notably, DCM will only find a difference in evidence for different models if they predict sufficiently distinct patterns of BOLD responses (Friston et al. 2003; Daunizeau et al. 2011). Temporal information to distinguish different models is limited in fMRI. Instead, fMRI-DCM mainly relies on condition-specific differences in the amplitudes of BOLD responses across regions (Stephan et al. 2010).

However, timing information is required to elucidate the precise time course of functional interactions between modality-specific and cross-modal areas (Hauk 2016). Therefore, future studies should employ methods with a high temporal resolution, such as electro- and magnetoencephalography to further investigate task-dependent functional and effective connectivity during conceptual processing. In particular, a high temporal resolution is necessary to determine whether modality-specific areas are engaged before, after, or simultaneously as cross-modal convergence zones (Kiefer et al. 2011). This question relates to the issue of bottom-up versus top-down information flow: A first engagement of modality-specific cortices would suggest bottom-up information flow, whereas an initial activation of cross-modal zones would indicate top-down processing (Fernandino et al. 2016). Timing information is also key to further refine theories of task dependency in conceptual processing. Specifically, it is currently unclear at which processing stage(s) conceptual processing is modulated by the task (Hauk 2016; but see Hoenig et al. 2008).

### A Refined Model of the Neural Architecture Underlying Conceptual Processing

Overall, our findings support theories that assume conceptual processing to rely on a flexible multilevel architecture grounded in the perceptual-motor systems (Binder and Desai 2011; Kemmerer 2015; Fernandino et al. 2016). For instance, we recently proposed that conceptual knowledge is supported by a representational hierarchy from modality-specific perceptual-motor regions via multimodal convergence zones (e.g., left PPC) to an amodal hub in the ATL (Kiefer and Harpaintner 2020; Kuhnke et al. 2020b). Moreover, we argued that this system is dynamic, with different regions being engaged depending on the task (Hoenig et al. 2008; Yee and Thompson-Schill 2016; Popp et al. 2019a).

Our model is related to two other prominent theories, the “hub-and-spokes” (Patterson et al. 2007; Lambon Ralph et al. 2016) and “embodied abstraction” (Binder and Desai 2011; Fernandino et al. 2016) models. Whereas the hub-and-spokes model posits that modality-specific “spoke” regions converge onto a single cross-modal “hub” in the ATL, the embodied abstraction model proposes a hierarchy of cross-modal convergence zones in the inferior parietal, temporal, and medial prefrontal cortices. In line with embodied abstraction, our model proposes multiple levels of cross-modal areas. Consistent with the hub-and-spokes model, it considers the ATL as the top-level, most abstract cross-modal hub. However, our model differs from both approaches in that it distinguishes among cross-modal convergence zones between “multimodal” regions (e.g., left PPC) that retain modality-specific information and the “amodal” ATL that does not.

We now refine this model in two ways: First, we subdivide modality-specific areas into multiple levels (Fig. 8*A*). As we found that not only high-level, but also low-level sensory-motor areas contribute to conceptual processing, we propose to subdivide modality-specific areas into low-level areas and “unimodal convergence zones” that contain more abstract, but still modality-specific representations (Damasio 1989; Mesulam 1998; Simmons and Barsalou 2003). Second, we add information about task-dependent functional coupling to the model (Fig. 8*B*). This picture illustrates that functional coupling in the conceptual system is extensive, involving interactions between various hierarchy levels. We found functional coupling between modality-specific and amodal regions (e.g., aIPL/S1 and ATL), modality-specific and multimodal regions (e.g., M1/S1 and PPC), multimodal and amodal regions (e.g., mPFC and ATL), different modality-specific regions (e.g., MFG/PreCS and auditory thalamus), and different multimodal regions (e.g., PPC and mPFC). We even found some evidence for coupling across modalities (e.g., PPC and visual cortex coupled during sound feature retrieval). Importantly, functional coupling is flexible and systematically depends on the task, similar to functional activation. Modality-specific regions selectively come into play when the knowledge they represent is task-relevant: Somatomotor regions show increased coupling selectively during action knowledge retrieval, and auditory regions during sound knowledge retrieval. The multimodal PPC acts as a functional coupling switchboard, flexibly adapting its connectivity profile to task-relevant modality-specific nodes. In contrast, the amodal ATL mainly shows task-dependent interactions with other high-level cross-modal hubs, with few connections to modality-specific cortices.

Our model is supported by a recent computational modeling study (Jackson et al. 2021), which revealed that the core functions of the conceptual system—conceptual abstraction and task dependency—are best achieved by a hierarchical multilevel architecture composed of a modality-specific layer, an intermediate layer (~multimodal regions), and a single top-level hub (~amodal ATL). In line with our findings, the optimal model exhibited connectivity between modality-specific and intermediate nodes, between intermediate nodes and the top-level hub, as well as sparse “shortcut” connections between the hub and modality-specific nodes.

## Notes

We thank Annika Tjuka for her tremendous help during data acquisition. We also thank Anke Kummer, Nicole Pampus, and Sylvie Neubert for acquiring participants and assisting the fMRI measurements. Moreover, we thank Toralf Mildner for implementing the dual-echo fMRI sequence. We are also grateful to Marie Beaupain and Maike Herrmann for their assistance in stimulus creation and piloting. Finally, we wish to thank two anonymous reviewers for their insightful comments, which contributed to a substantial improvement of this manuscript. *Conflict of Interest*: None declared.

## Funding

Max Planck Society; German Research Foundation (HA 6314/3-1, HA 6314/4-1 to G.H.).

## Supplementary Material

Kuhnke2021_CerCor_Supplementary_bhab026
