# Supplementary material for: Task-Dependent Functional and Effective Connectivity during Conceptual Processing
Source: Cereb Cortex. 2021 Mar 3;31(7):3475–93. doi: 10.1093/cercor/bhab026 (PMC8196308; doi:10.1093/cercor/bhab026)
Supplement: Kuhnke2021_CerCor_Supplementary_bhab026 [file kuhnke2021_cercor_supplementary_bhab026.pdf]

Supplementary Material

Kuhnke et al. (2021) *Cerebral Cortex*

"Task-dependent functional and effective connectivity during conceptual processing"

Supplementary Materials and Methods

Temporal Signal-to-Noise Ratio (tSNR)

We employed a dual-echo sequence to minimize susceptibility artifacts and maximize BOLD sensitivity throughout the entire brain (Poser et al. 2006; Halai et al. 2014), including in regions suffering from signal dropout in single-echo EPI, such as the anterior temporal lobe (ATL) (Devlin et al. 2000). To assess the signal quality in the ATL and other regions, we computed the temporal signal-to-noise ratio (tSNR) by dividing the mean signal in each voxel by the standard deviation of the voxel's signal over time (Friedman et al. 2006; Fairhall and Caramazza 2013). While signal quality was generally higher in regions outside vs. inside the ATL (Table S1), tSNR within the ATL was clearly above 20, which is considered the threshold for signal detection at 3T (Friedman et al. 2006; Binder et al. 2011).

Table S1. Temporal signal-to-noise ratio (tSNR) in anatomical regions-of-interest (ROIs). Bilateral ROIs were extracted from the Harvard-Oxford atlas (http://www.fmrib.ox.ac.uk/fsl/) and thresholded at 30% probability for a voxel to belong to a region.

**Region**	**mean tSNR (SD)**	**Region**	**mean tSNR (SD)**	**Region**	**mean tSNR (SD)**
**Anterior temporal lobe (ATL)**		**Posterior temporal lobe**		**Other regions**	
Temporal pole	61.62 (17.96)	Heschl's gyrus	80.93 (14.31)	AG	122.89 (17.59)
aSTG	85.89 (12.73)	pSTG	101.54 (13.47)	Occipital pole	108.39 (27.56)
aMTG	79.79 (9.63)	pMTG	94.36 (14.95)	Postcentral gyrus	120.16 (19.78)
aITG	62.99 (10.5)	pITG	67.61 (16.96)	aIFG	99.0 (30.36)
aFG	50.53 (12.64)			pIFG	103.26 (10.05)

STG = superior temporal gyrus; MTG = middle temporal gyrus; ITG = inferior temporal gyrus; FG = fusiform gyrus; AG = angular gyrus; IFG = inferior frontal gyrus; a (prefix) = anterior; p (prefix) = posterior.

Supplementary Results

Psychophysiological interactions (PPI)

"Amodal seed" (left ATL)

As the anterior temporal lobe (ATL) is widely considered a central, amodal "hub" of the conceptual system (Patterson et al. 2007; Jefferies 2013; Lambon Ralph et al. 2016), we performed a supplementary PPI analysis seeding in the amodal ATL. The "amodal seed" was defined as the ATL region most strongly engaged for "general" conceptual processing, using the contrast [lexical decision: words > pseudowords]. This region was located in the left ATL (containing parts of the anterior superior, middle, and inferior temporal gyri). All other methods were identical to our other PPI analyses (see Materials and Methods).

We found that during sound judgments, sound feature retrieval (high > low sound words) significantly increased coupling between the amodal seed (left ATL) and bilateral precuneus / posterior cingulate cortex (PC/PCC) (Figure S1A; Table S10). This PC/PCC region did not overlap with the auditory localizer (Figure S1B), indicating that it constitutes a higher-level, cross-modal region. Interaction analyses further revealed a significant TASK x SOUND interaction in bilateral PC/PCC, driven by a stronger coupling increase for sound features (high vs. low sound words) during sound judgments than during action judgments and lexical decisions (Figure S1C; Table S11). Action features (high > low action words) did not induce significant coupling changes during sound judgments.

During action judgments, we found no significant effects that survived stringent multiple comparisons correction (voxel-wise $p < 0.001$, cluster-wise $p < 0.05$ FWE-corrected). However, an exploratory analysis at $p < 0.001$ uncorrected (extent > 10 voxels) showed that action feature retrieval (high > low action words) selectively increased coupling between the amodal seed (left ATL) and the left dmPFC (Figure S2A; Table S12). Left dmPFC did not overlap with the somatomotor localizer (Figure S2B), indicating that it represents a higher-level, cross-modal area. Interaction analyses revealed a TASK x ACTION interaction within left dmPFC, which was driven by a larger coupling increase for action features (high vs. low action words) during action judgments than sound judgments and lexical decisions (Figure S2C; Table S13). Sound features (high > low sound words) did not increase coupling during action judgments (even at $p < 0.001$ uncorrected).

We found no significant effects during lexical decisions (even at $p < 0.001$ uncorrected).

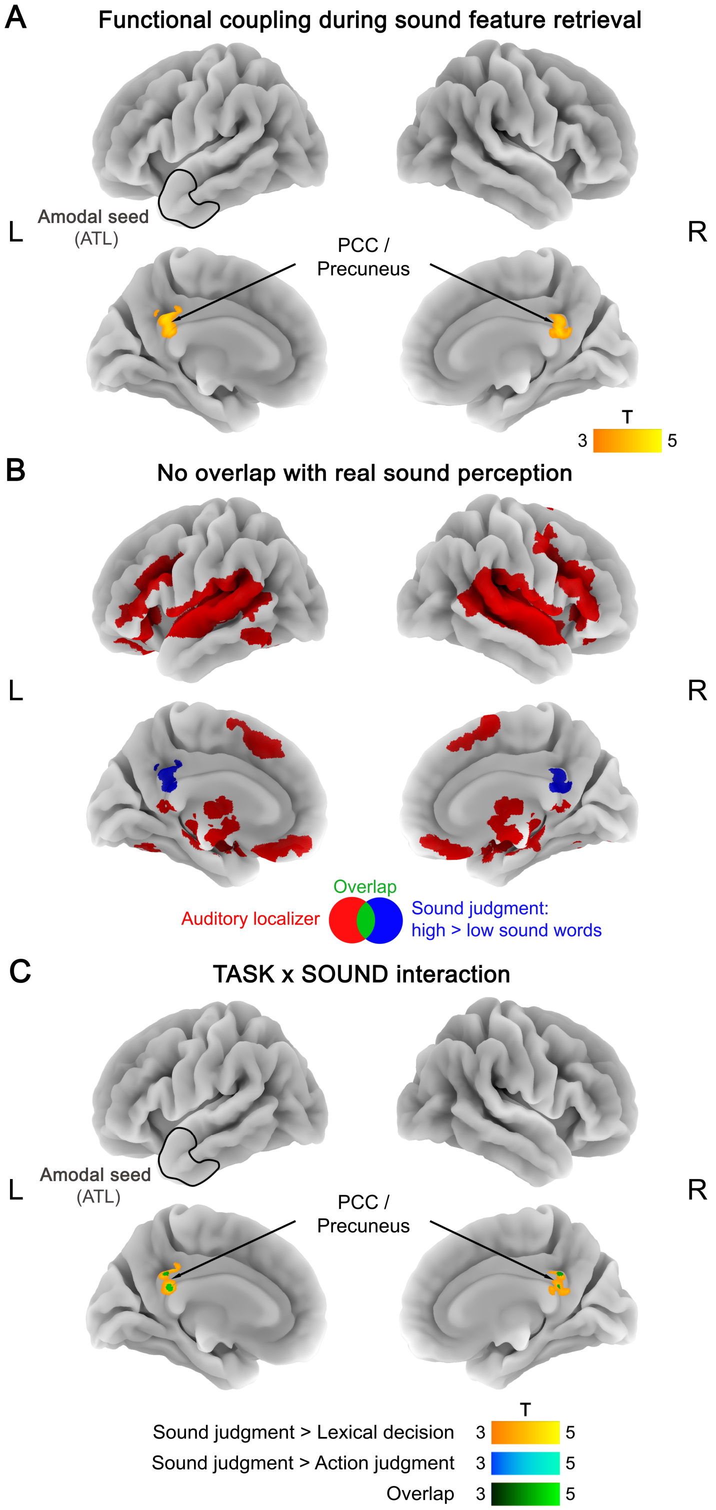

Figure S1. (A) Functional coupling with the amodal seed (left ATL) during sound feature retrieval (sound judgments: high > low sound words). (B) No overlap between functional coupling with the amodal seed during sound feature retrieval (blue) and activation for the auditory localizer (red; real sounds > silence). (C) TASK x SOUND interaction in functional coupling with the amodal seed, reflecting a stronger coupling increase for sound features (high vs. low sound words) during sound judgments than during lexical decisions (yellow), action judgments (blue), or both (green). All statistical maps were thresholded at a voxel-wise $p < 0.001$ and a cluster-wise $p < 0.05$ FWE-corrected.

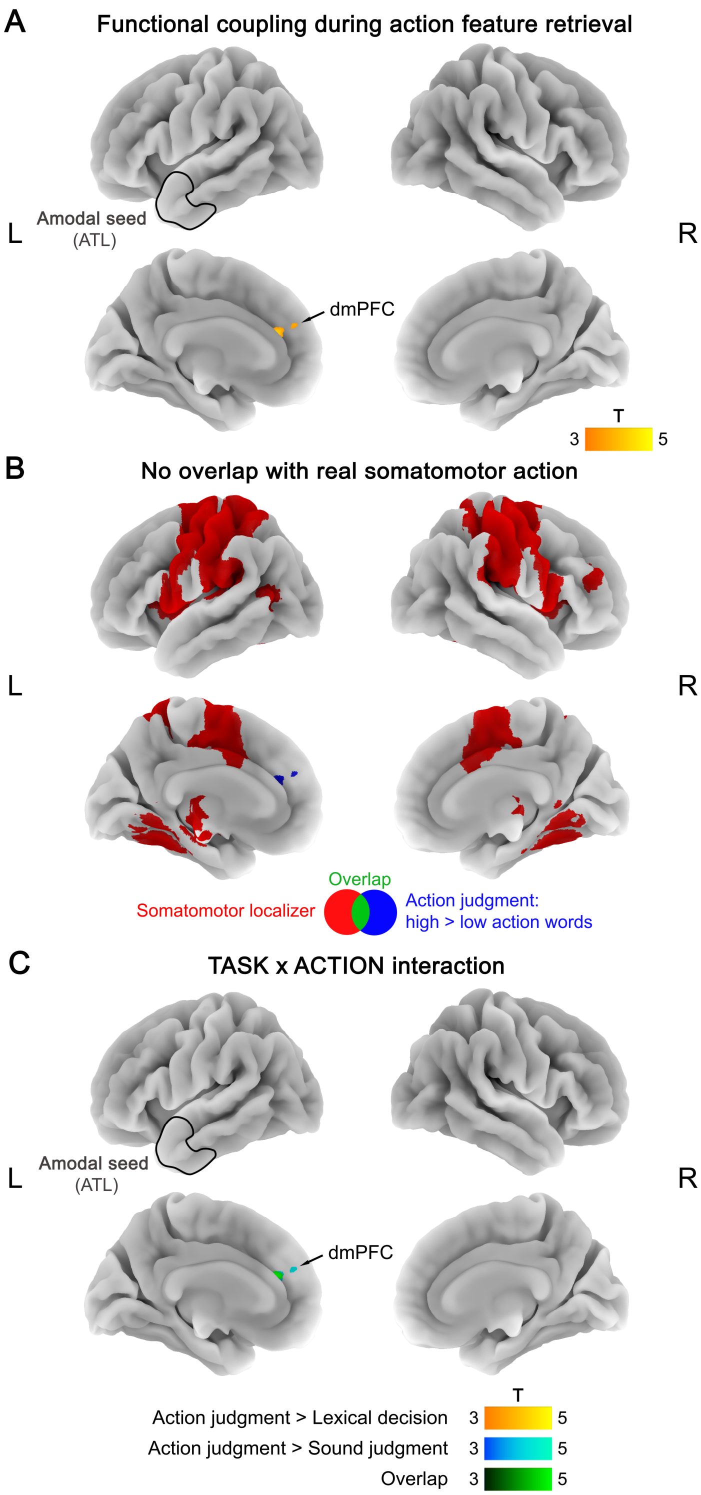

Figure S2. (A) Functional coupling with the amodal seed (left ATL) during action feature retrieval (action judgments: high > low action words). (B) No overlap between functional coupling with the amodal seed during action feature retrieval (blue) and activation for the somatomotor localizer (red; hand movements > rest). (C) TASK x ACTION interaction in functional coupling with the amodal seed, reflecting a stronger coupling increase for action features (high vs. low action words) during action judgments than during lexical decisions (yellow), sound judgments (blue), or both (green). Note that these results come from an exploratory analysis thresholded at $p < 0.001$ uncorrected (extent > 10 voxels).

Ratings–RT correlations

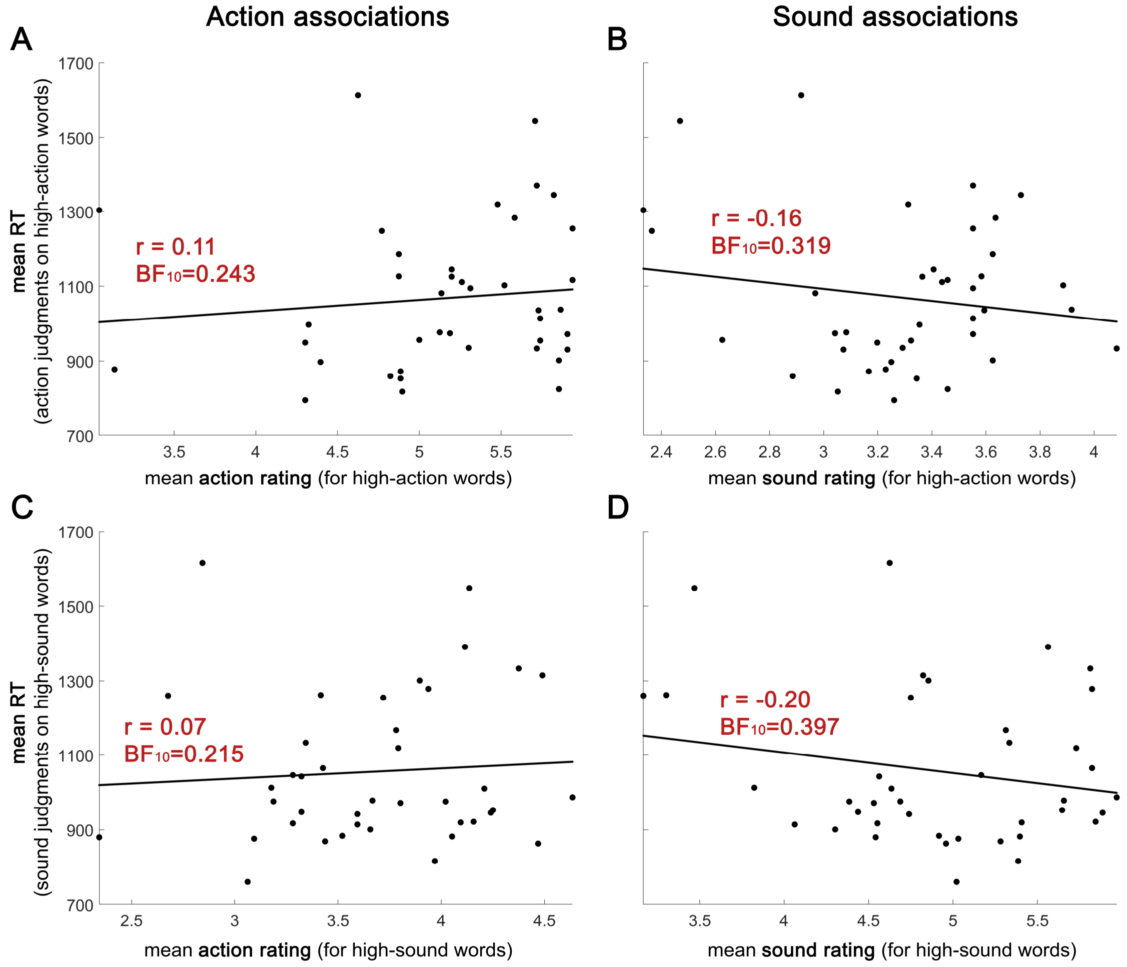

Figure S3. Mean response times (RTs) for action judgments on high-action words were not associated with the mean action (A) or sound (B) ratings for the same words. Similarly, mean RTs for sound judgments on high-sound words were not associated with the mean sound (D) or action (C) ratings for these words.

Tables of coordinates

The following tables report brain regions showing task-dependent functional coupling with a certain seed region during conceptual processing. Coordinates are in MNI space. Up to 3 peaks per cluster are reported (> 8 mm apart). AAC = auditory association cortex; ATL = anterior temporal lobe; dmPFC = dorsomedial prefrontal cortex; FG = fusiform gyrus; IFG = inferior frontal gyrus; IPL = inferior parietal lobe; ITG = inferior temporal gyrus; LOC = lateral occipital cortex; MCC = middle cingulate cortex; MTG = anterior middle temporal gyrus; M1 = primary motor cortex; PCC = posterior cingulate cortex; PPC = posterior parietal cortex; SPL = superior parietal lobe; S1 = primary somatosensory cortex; SPL = superior parietal lobe; STS = posterior superior temporal sulcus; a (prefix): anterior; p (prefix): posterior; L = left; R = right.

Table S2. Brain regions showing functional coupling with the motor seed (left aIPL/S1) during action feature retrieval.

Region	Cluster size (mm^3)	x	y	z	T
Action judgments: *high > low action words*					
L ATL	734				
L aITG		-62	-17	-25	4.57
L aMTG		-64	-10	-22	4.15
L aITG		-60	-27	-28	3.94

Table S3. TASK x ACTION interaction in functional coupling with the motor seed (left aIPL/S1).

Region	Cluster size (mm^3)	x	y	z	T
Action judgment > lexical decision for high vs. low action words					
L ATL (aITG)	94	-60	-27	-28	3.82
L ATL (aMTG)	31	-67	-14	-25	3.45

Table S4. Brain regions showing functional coupling with the auditory seed (left MFG/PreCS) during sound feature retrieval.

Region	Cluster size (mm^3)	x	y	z	T
(A) Sound judgments: high > low sound words					
L thalamus	1781				
L thalamus (temporal)		-4	-22	12	4.91
L thalamus (prefrontal)		-17	-17	12	4.79
L thalamus (parietal)		-17	-22	15	4.76
R SPL	750				
R SPL (7PC)		23	-54	58	4.66
R SPL (7A)		18	-62	55	4.52

Region	Cluster size (mm³)	x	y	z	T
R SPL (5L)		13	-50	58	3.52
R FG	703				
R FG2		-42	-70	-10	4.49
R FG4		-40	-60	-10	4.39
R FG2		-37	-67	-12	4.06
(B) Overlap with auditory localizer					
L thalamus	156				
L thalamus (temporal)		-7	-14	12	3.72
L thalamus (prefrontal)		-14	-10	12	3.52

Table S5. TASK x SOUND interaction in functional coupling with the auditory seed (left MFG/PreCS).

Region	Cluster size (mm³)	x	y	z	T
(A) Sound judgment > lexical decision for high vs. low sound words					
R SPL	531				
R SPL (7PC)		23	-54	58	4.79
R SPL (7A)		23	-60	58	4.68
R SPL (7A)		16	-60	58	4.01
L FG (FG4)	94	-40	-60	-10	4.01
L Thalamus (prefrontal)	47	-4	-20	8	3.94
L Thalamus (parietal)	31	-17	-22	12	3.43
L FG (FG2)	31	-34	-67	-12	3.83
(B) Sound judgment > action judgment for high vs. low sound words					
L FG	266				
L FG2	0	-42	-67	-10	4.62
L FG4	0	-40	-62	-10	4.28
R SPL (7A)	156	20	-57	58	3.95
(C) Overlap					
R SPL (7A)	156	20	-57	58	3.95

Table S6. Brain regions showing functional coupling with the multimodal seed (left PPC) during action feature retrieval.

Region	Cluster size (mm³)	x	y	z	T
(A) Action judgments: high > low action words					
R pSTS	1156				
R pSTS		50	-42	15	4.56
R pSTS		58	-44	5	4.07
L M1 / SPL	1000				

Region	Cluster size (mm³)	x	y	z	T
L SPL (5L)		-12	-50	75	4.33
L M1 (4a)		-7	-40	80	4.04
L M1 (4a)		-10	-37	72	4.00
L M1 / S1	828				
L M1 (4a)		-22	-30	58	4.26
L M1		-30	-24	55	4.10
L S1 (3b)		-44	-22	58	3.82
(B) Overlap with motor localizer					
L M1 / S1	766				
L M1		-30	-24	55	4.10
L M1 (4p)		-24	-30	58	3.85
L S1 (3b)		-44	-22	58	3.82
L M1 (4a)	188	-20	-37	72	3.86

Table S7. TASK x ACTION interaction in functional coupling with the multimodal seed (left PPC).

Region	Cluster size (mm³)	x	y	z	T
(A) Action judgment > lexical decision for high vs. low action words					
R pSTS	156	60	-44	2	4.10
(B) Action judgment > sound judgment for high vs. low action words					
R pSTS	844				
R pSTS		58	-47	10	4.58
R pSTS		56	-44	8	4.58
L M1/S1	172				
L M1 (4a)		-12	-40	72	3.84
L S1 (3b)		-17	-40	70	3.59
L M1	125				
L M1 (4a)		-40	-24	58	3.92
L M1 (4a)		-40	-24	65	3.47
L M1	94				
L M1 (4a)		-14	-32	80	3.99
L M1 (4a)		-20	-34	78	3.84
(C) Overlap					
R pSTS	156	60	-44	2	4.01

Table S8. Brain regions showing functional coupling with the multimodal seed (left PPC) during sound feature retrieval.

Region	Cluster size (mm³)	x	y	z	T
(A) Sound judgments: high > low sound words					
L/R precuneus / medial SPL / MCC	11016				
R precuneus		3	-47	48	5.38
R SPL (5Ci)		10	-32	40	5.20
R MCC		3	-42	42	5.07
L/R dmPFC / MCC	10891				
L dmPFC		0	8	45	5.58
L MCC		0	-14	40	5.26
L MCC		-2	18	35	5.05
L cerebellum / high-level visual cortex	2016				
L cerebellum (lobule VI)		-2	-70	-12	4.85
L cerebellum (lobule VI)		-17	-72	-25	4.51
L V4 (hOc4v)		-20	-72	-15	4.49
L S1	1688				
L S1 (area 2)		-44	-37	58	4.75
L S1 (area 2)		-47	-34	55	4.47
L S1 (area 3b)		-42	-24	48	4.24
L/R early visual cortex	1641				
R V1 (hOc1)		8	-64	5	4.70
L V1 (hOc1)		-12	-82	10	4.08
R V2 (hOc2)		6	-74	12	4.01
R high-level visual cortex	1578				
R pITG		56	-62	-10	4.54
R pITG (hOc4la)		56	-64	-5	4.49
R pMTG		50	-44	-5	4.35
R LOC	1531				
R LOC (middle occipital)		38	-72	28	4.50
R LOC (middle occipital)		40	-70	25	4.29
R LOC (middle occipital)		33	-70	35	3.76
L/R thalamus	1516				
L thalamus (prefrontal)		-10	-12	10	5.01
R thalamus (temporal)		6	-24	15	4.57
L thalamus (prefrontal)		-7	-22	8	4.32
R IPL	1453				
R IPL (PFcm)		60	-30	25	4.85
R IPL (PFop)		58	-20	25	4.42
R IPL (PF)		68	-27	28	4.28
R dmPFC	1344				
R dmPFC (SFG)		20	10	65	4.54
R dmPFC (SFG)		18	6	70	4.06
R dmPFC (SFG)		16	0	72	3.96
L LOC	1125				

Region	Cluster size (mm³)	x	y	z	T
L LOC (middle occipital)		-24	-74	30	4.33
L LOC (superior occipital)		-24	-67	32	4.20
L precuneus		-14	-70	30	3.97
R IPL	938				
R IPL (PFm)		60	-47	32	4.46
R IPL (PFm)		50	-44	30	4.02
R IPL (PF)		63	-37	38	3.94
L LOC	891				
L LOC (middle occipital)		-27	-80	12	4.65
L LOC (hOc4lp)		-32	-90	8	4.05
L LOC (hOc4lp)		-34	-87	10	3.89
L AAC / IFG	844				
L AAC (TE 3)		-52	10	-8	5.13
L IFG (pars triangularis)		-47	20	-2	3.49
(B) Overlap with auditory localizer					
R IPL (PFcm)	594				
R IPL (PFcm)		60	-30	25	4.55
R IPL (PFcm)		50	-32	22	4.09
L thalamus	391				
L thalamus (prefrontal)		-10	-12	10	4.57
L thalamus (prefrontal)		-14	-10	10	4.20
L/R dmPFC	344				
L/R dmPFC		0	8	58	3.94
R dmPFC		3	0	60	3.52
L AAC (TE 3)	344	-52	10	-8	5.13
L/R dmPFC	281	0	26	48	4.10
R thalamus (prefrontal)	234	10	-14	10	4.29
L cerebellum (lobule VI)	219	-24	-67	-22	4.32

Table S9. TASK x SOUND interaction in functional coupling with the multimodal seed (left PPC).

Region	Cluster size (mm³)	x	y	z	T
(A) Sound judgment > lexical decision for high vs. low sound words					
R SPL / IPS	3047				
R SPL (7PC)		28	-52	55	5.66
R SPL (7A)		23	-64	58	5.65
R precuneus		13	-60	55	5.54
R high-level visual cortex	1422				
R LOC (hOc4la)		48	-74	-5	4.84
R ITG		53	-54	-10	4.81
R LOC (hOc4la)		50	-80	-8	4.61
R MCC / precuneus	1375				

Region	Cluster size (mm³)	x	y	z	T
R MCC		3	-42	42	4.74
R MCC (5M)		6	-37	50	4.11
R precuneus (5Ci)		8	-44	50	4.09
L S1	1297				
L S1 (area 1)		-32	-42	65	4.94
L S1 (area 3b)		-42	-22	50	4.66
L S1 (area 2)		-40	-42	55	4.30
R LOC	750				
R LOC (superior occipital)		30	-74	40	5.40
R LOC (middle occipital)		36	-72	25	3.73
L MCC	719				
L MCC		0	8	32	4.33
L MCC		-2	16	35	4.29
R MCC		6	16	38	3.92
L SPL	609				
L SPL (5L)		-17	-42	75	5.10
L Paracentral Lobule		-10	-37	70	4.83
L Paracentral Lobule		-10	-40	78	4.45
L LOC / precuneus	484				
L LOC (middle occipital)		-24	-74	30	5.13
L precuneus		-14	-70	32	3.91
L LOC (superior occipital)		-17	-77	30	3.89
L LOC	453				
L LOC (middle occipital)		-34	-80	10	4.23
L LOC (hoc4la)		-40	-82	10	4.06
L LOC (hoc4lp)		-32	-90	8	3.82
R MCC	422	10	-34	35	5.31
R early visual cortex	422				
R V1 (hOc1)		8	-70	5	4.01
R V2 (hOc2)		6	-74	12	3.93
L/R dmPFC	375				
R dmPFC		6	0	62	4.06
L/R dmPFC		0	6	58	3.74
R dmPFC		3	-4	60	3.73
L AAC (TE 3)	328	-52	10	-5	4.54
L/R dmPFC	297				
R dmPFC		3	-4	70	4.22
L dmPFC		-7	-2	70	4.18
L MCC	297				
L MCC		-7	-32	35	4.24
L MCC		-12	-24	38	4.18
L MCC		-4	-34	38	3.71
L MCC	281				
L MCC		-7	-10	45	4.09

Region	Cluster size (mm³)	x	y	z	T
L MCC		-10	-2	42	3.72
R IPL	266				
R IPL (PFt)		58	-17	30	3.77
R IPL (PFt)		56	-20	32	3.64
R IPL (PFop)		63	-24	25	3.59
(B) Sound judgment > action judgment for high vs. low sound words					
R SPL / IPS	1359				
R SPL (7A)		26	-64	55	4.90
R SPL (7PC)		30	-50	55	4.12
R IPS (hIP3)		28	-57	52	4.07
L LOC (middle occipital)	469	-27	-67	30	4.69
(C) Overlap					
R SPL / IPS	1281				
R SPL (7A)		26	-64	55	4.65
R IPS (hIP3)		28	-57	52	4.07
R SPL (7PC)		30	-50	55	4.02
L LOC (middle occipital)	125	-27	-72	28	3.83

Table S10. Brain regions showing functional coupling with the amodal seed (left ATL) during sound feature retrieval.

Region	Cluster size (mm³)	x	y	z	T
Sound judgments: high > low sound words					
L/R Precuneus/PCC	1094				
R PCC		8	-44	22	4.44
L PCC		-2	-44	22	4.10
R Precuneus		6	-50	20	3.93

Table S11. TASK x SOUND interaction in functional coupling with the amodal seed (left ATL).

Region	Cluster size (mm³)	x	y	z	T
(A) Sound judgment > lexical decision for high vs. low sound words					
L PCC	219	-4	-44	22	4.71
L/R MCC	188				
L MCC		-2	-44	35	4.00
L/R MCC		0	-47	32	3.66
R Precuneus	109	6	-52	20	3.80

Region	Cluster size (mm³)	x	y	z	T
(B) Sound judgment > action judgment for high vs. low sound words					
L/R MCC	16	0	-47	32	3.82
L PCC	16	-2	-44	22	3.44

Table S12. Brain regions showing functional coupling with the amodal seed (left ATL) during action feature retrieval.

Region	Cluster size (mm³)	x	y	z	T
Action judgments: high > low action words					
L dmPFC	156	-12	48	25	4.34
L dmPFC	156	-12	38	22	4.25

Note that these results come from an exploratory analysis at $p < 0.001$ uncorrected (extent > 10 voxels).

Table S13. TASK x ACTION interaction in functional coupling with the amodal seed (left ATL).

Region	Cluster size (mm³)	x	y	z	T
(A) Action judgments > lexical decisions for high vs. low action words					
L dmPFC	78	-12	38	22	4.12
L dmPFC	16	-12	46	25	3.55
(B) Action judgments > sound judgments for high vs. low action words					
L dmPFC	156	-14	36	22	5.33
L dmPFC	109	-10	48	25	4.29
